# Mutation of the Diamond-Blackfan Anemia Gene *Rps7* in Mouse Results in Morphological and Neuroanatomical Phenotypes

**DOI:** 10.1371/journal.pgen.1003094

**Published:** 2013-01-31

**Authors:** Dawn E. Watkins-Chow, Joanna Cooke, Ruth Pidsley, Andrew Edwards, Rebecca Slotkin, Karen E. Leeds, Raymond Mullen, Laura L. Baxter, Thomas G. Campbell, Marion C. Salzer, Laura Biondini, Gretchen Gibney, Françoise Phan Dinh Tuy, Jamel Chelly, H. Douglas Morris, Johannes Riegler, Mark F. Lythgoe, Ruth M. Arkell, Fabrizio Loreni, Jonathan Flint, William J. Pavan, David A. Keays

**Affiliations:** 1 National Human Genome Research Institute, National Institutes of Health, Bethesda, Maryland, United States of America; 2 Wellcome Trust Centre for Human Genetics, University of Oxford, Oxford, United Kingdom; 3 Institute of Molecular Pathology, Vienna, Austria; 4 Department of Biology, University of Rome Tor Vergata, Roma, Italy; 5 Institut Cochin, Université Paris Descartes, CNRS (UMR 8104), Paris, France; 6 National Institute of Neurological Disorders and Stroke, National Institutes of Health, Bethesda, Maryland, United States of America; 7 Centre for Advanced Biomedical Imaging, Department of Medicine and Institute of Child Health, University College London, London, United Kingdom; 8 Early Mammalian Development Laboratory, Research School of Biology, College of Medicine, Biology, and Environment, Australian National University, Canberra, Australia; Stanford University School of Medicine, United States of America

## Abstract

The ribosome is an evolutionarily conserved organelle essential for cellular function. Ribosome construction requires assembly of approximately 80 different ribosomal proteins (RPs) and four different species of rRNA. As RPs co-assemble into one multi-subunit complex, mutation of the genes that encode RPs might be expected to give rise to phenocopies, in which the same phenotype is associated with loss-of-function of each individual gene. However, a more complex picture is emerging in which, in addition to a group of shared phenotypes, diverse RP gene-specific phenotypes are observed. Here we report the first two mouse mutations (*Rps7^Mtu^* and *Rps7^Zma^*) of ribosomal protein S7 (*Rps7*), a gene that has been implicated in Diamond-Blackfan anemia. *Rps7* disruption results in decreased body size, abnormal skeletal morphology, mid-ventral white spotting, and eye malformations. These phenotypes are reported in other murine RP mutants and, as demonstrated for some other RP mutations, are ameliorated by *Trp53* deficiency. Interestingly, *Rps7* mutants have additional overt malformations of the developing central nervous system and deficits in working memory, phenotypes that are not reported in murine or human RP gene mutants. Conversely, *Rps7* mouse mutants show no anemia or hyperpigmentation, phenotypes associated with mutation of human *RPS7* and other murine RPs, respectively. We provide two novel RP mouse models and expand the repertoire of potential phenotypes that should be examined in RP mutants to further explore the concept of RP gene-specific phenotypes.

## Author Summary

Ribosomes are composed of two subunits that each consist of a large number of proteins, and their function of translating mRNA into protein is essential for cell viability. Naturally occurring or genetically engineered mutations within an individual ribosomal protein provide a valuable resource, since the resulting abnormal phenotypes reveal the function of each ribosomal protein. A number of mutations recently identified in mammalian ribosomal subunit genes have confirmed that homozygous loss of function consistently results in lethality; however, haploinsufficiency causes a variety of tissue-specific phenotypes. In this paper, we describe the first mutant alleles of the gene encoding ribosomal protein S7 (*Rps7*) in mouse. *Rps7* haploinsufficiency causes decreased size, abnormal skeletal morphology, mid-ventral white spotting, and eye malformations, phenotypes that also occur with haploinsufficiency for other ribosomal subunits. Additionally, significant apoptosis occurs within the developing central nervous system (CNS) along with subtle behavioral phenotypes, suggesting RPS7 is required for CNS development. Mutation of human *RPS7* has been implicated in Diamond-Blackfan anemia (DBA), yet the murine alleles do not present an analogous phenotype. The phenotypes we observe in the *Rps7* mouse mutants indicate RPS7 should be considered as a candidate for a broader spectrum of human diseases.

## Introduction

Ribosomes are responsible for constructing the myriad proteins required for the function of each individual cell. Ribosomes themselves, which consist of small (40S) and large (60S) subunits, are assembled from about 80 different ribosomal proteins (RPs) along with four species of rRNA synthesized in the nucleolus [1], [2]. RPs fall broadly into two categories; the RPL proteins that make up the large ribosomal subunit, and the RPS proteins that constitute the small subunit. Mutations in both RPL and RPS genes have been implicated in a set of shared phenotypic characteristics in invertebrates. This is best illustrated by the approximately 50 *Drosophila melanogaster Minute* mutants, a collection of ribosomal gene mutations that are characterized by developmental delay, short thin bristles, growth retardation, reduced fertility and recessive lethality [3].

In vertebrates, a phenotypic overlap among RP mutants occurs similar to that seen in *Drosophila Minute* mutants, however additional phenotypic complexity is emerging, in which mutation or suppression of RPs results in some phenotypes that depend upon which gene is mutated. This is highlighted by a recent study in which 20 different RP genes were targeted using morpholino antisense oligos in the zebrafish *Danio rerio* [4]. Whereas some phenotypes were shared among knockdowns, such as hypoplasia of the yolk sac, others were gene-specific. For instance, knockdown of *rps15* resulted in an enlarged 4^th^ ventricle of the brain, whereas knockdown of *rpl35a* caused a sharply bent tail, and targeting of *rps29* produced an enlarged lens [4]. A similar picture of phenotypic overlap paired with gene-specific phenotypes is emerging in mice. For example, mutations in *Rps19* and *Rps20* result in increased epithelial pigmentation, a ventral belly spot, small body size, and a reduction in red blood cell count [5]. Mice with mutations in *Rpl24* also have ventral belly spots and body size reduction, but they present with additional retinal abnormalities and skeletal defects [6]. Mice harboring the *Rpl27a* sooty foot allele share an epidermal hyperpigmentation phenotype with *Rps19* and *Rps20* mutants, but display the additional feature of cerebellar ataxia [7]. These distinct features of individual RP mutant phenotypes suggest that vertebrate RPs may have unique, tissue-specific functions and/or tissue-specific expression levels. Indeed, the broad spectrum of distinct phenotypes that have been characterized in a relatively small number of mammalian RP mutants also hints at possible extra-ribosomal functions of RPs.

In humans, mutations in RPs have been implicated in hematopoetic disorders, most notably Diamond-Blackfan anemia (DBA), which has been attributed to mutations in *RPS19*, *RPS26*, *RPS24*, *RPS17*, *RPS10*, *RPS7*, *RPL35a*, *RPL26*, *RPL11*, and *RPL5* [8]–[14]. While anemia is a shared phenotype among all patients carrying these various gene mutations, some specific attributes are associated with individual genes. For example, mutations in *RPL5* are associated with cleft palate and anomalies of the thumb and heart, whereas isolated thumb malformations are predominant in patients carrying mutations in *RPL11* [11].

In this paper, we further examine the role of ribosomes in mammalian development by investigating *Rps7*. Through analysis of two new ENU-induced mouse mutants of *Rps7*, montu (*Mtu*) and zuma (*Zma*), we show that mutation of *Rps7* impairs ribosomal biogenesis, resulting in variable lethality and reduced body size accompanied by abnormal skeletal, melanocyte, eye, and central nervous system (CNS) development. While many of these phenotypes have previously been associated with mutation of murine RP genes, the findings of overt brain malformations and behavioral abnormalities are novel. Similar to mutation of other RP genes in the mouse, the penetrance of the *Rps7-*associated phenotypes is affected by genetic background and the overt phenotypes are suppressed by TRP53 deficiency. These mutants provide the first mouse models of *Rps7* disruption and increase our understanding of the phenotypic consequences of mammalian RP mutations.

## Results

### Identification and genetic mapping of montu and zuma

**Figure 1:**
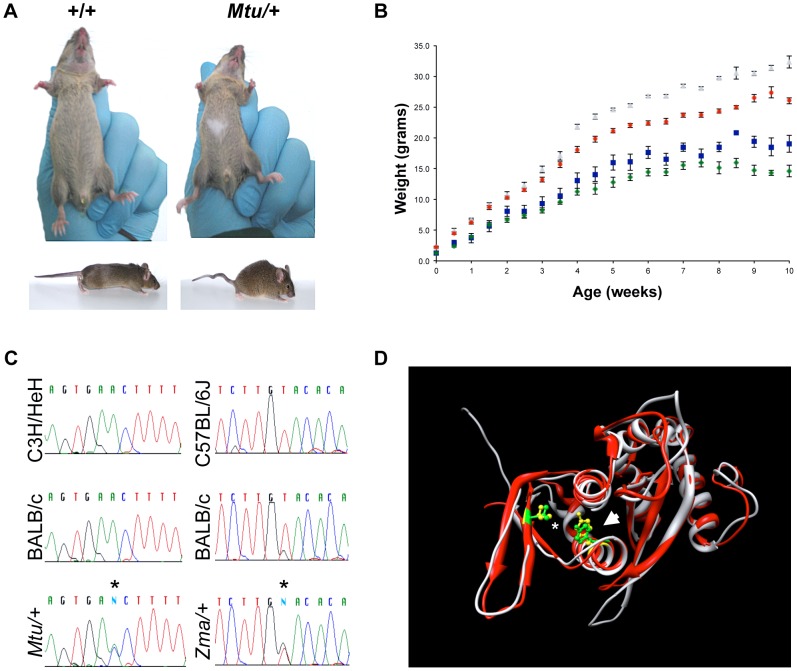
Heterozygous mutation of *Rps7* results in visible white spotting, small body size, and tail kinking. (A) Montu (*Mtu*) heterozygous mice exhibiting a white belly spot and kinked tail were identified in an ENU mutagenesis screen. An independent ENU screen identified zuma (*Zma*) mice with similar phenotypes (data not shown). (B) Heterozygote *Mtu/+* male (blue) and female (green) mice have a significantly reduced body weight compared to wild-type male (gray) and female (red) littermates. (C) Sequencing revealed novel *Rps7* point mutations in *Mtu/+* (c.574T>G, encoding p.V156G) and *Zma/+* (c.637A>C, encoding p.Y177S) mice. Sequence traces shown are for the antisense strand. Detailed *Rps7* genomic structure information can be found at http://www.ncbi.nlm.nih.gov/gene/20115. (D) The predicted structural locations of mutated amino acids in *Rps7^Mtu^* and *Rps7^Zma^* alleles. The three-dimensional structures of RPS7 orthologs from *S. cerevisiae* (PDB ID 3U5C_H, red) and *T. thermophila* (PDB ID 2XZM_3, gray) are superimposed. The locations of the residues homologous to mouse p.V156 (*) and p.Y177 (arrow) are shown in green (*S. cerevisiae*) and in yellow (*T. thermophila*). Image generated with UCSF Chimera.

The montu *(Mtu)* mouse was identified in a large-scale ENU mutagenesis program [15], [16] exhibiting dominant inheritance of a ventral belly spot, kinked tail, and reduced body weight (Figure 1A, 1B). Linkage analysis was performed using a cross of the *Mtu* founder (BALB/c OlaHsd background) with C3H/HeH. Analysis of genotypes of 104 offspring demonstrated linkage to a 74 Mb region of proximal chromosome 12 that was further refined to a 3.43 Mb critical region (Figure S1A, S1C). DNA sequencing of exons and flanking regions of 17 candidate genes using genomic DNA from four affected animals and controls identified a single heterozygous sequence variant occurring only in affected mice within exon 6 of *Rps7*, which encodes a 194 amino acid ribosomal protein, RPS7 (or S7e, the eukaryotic specific homolog of the yeast S7A and S7B). The identified *Rps7* variant, c.574T>G (NM_011300), encodes a Gly substitution of a highly conserved Val residue (p.V156G; NP_035430) (Figure 1C, Figure S2A). Subsequent sequence analysis of 91 animals from a C3H/HeH congenic *Mtu* colony showed 100% concordance between the c.574T>G mutation and the affected phenotypes. Analysis of *Mtu* animals demonstrated variable penetrance on a mixed background, however 100% penetrance of the tail kink, belly spot, and small body size phenotypes when assessed in the C3H/HeH congenic colony. We also observed a dominant lethality phenotype with incomplete penetrance, 72% viability in F1 BALB/c:C3H mice (N = 251) and 26% viability in the C3H/HeH congenic colony (N = 260).

The zuma (*Zma*) mouse was independently identified as part of a sensitized ENU screen designed to identify mutations that increased the severity of neural crest defects observed in *Sox10* haploinsufficient mice (*Sox10^LacZ/+^*), a well characterized neural crest mutant which presents with a high frequency of small, white belly spots [17]. Affected backcross mice (BALB/cJ×C57BL/6J) from the *Zma* pedigree exhibited large white belly spots, tail kinks, and reduced body size. Linkage analysis of twelve affected N1 mice initially detected linkage of *Zma* to a region of chromosome 12 overlapping where the *Mtu* mutation was localized (Figure S1B, S1C). Subsequent sequencing of *Rps7* in *Zma* mice revealed a heterozygous A to C point mutation in exon 7 of *Rps7*, predicted to cause substitution of a conserved amino acid (p.Y177S; c.637A>C) (Figure 1C, Figure S2A). Genotyping analysis of *Zma* mice outcrossed to C57BL/6J to establish a congenic colony showed that the c.637A>C mutation was observed in 100% of affected mice. We initially observed incompletely penetrant phenotypes in N2 heterozygous *Zma* mice on a mixed BALB/cJ; C57Bl/6J background, where *Zma/+* mice showed 74% viability, 76% belly spots, and 90% tail kinks (N = 46). However, heterozygosity for the *Zma* allele rapidly changed to a completely penetrant, lethal phenotype during outcrossing onto C57BL/6J, and no *Zma/+* mice were observed at N4. This contrasted with the phenotype we observed during establishment of a congenic C3H/HeJ *Zma* colony; on this genetic background, the N3 generation exhibited a low frequency of belly spotting and tail kinks (4% and 0%, respectively; N = 41) yet no lethality, as *Zma* heterozygotes were observed at the expected frequency through N6. The comparably mild phenotype we observed in the congenic C3H/HeJ *Zma* colony suggests that the *Mtu* allele may exert more severe phenotypic effects than the *Zma* allele. This hypothesis is supported by the observations that *Mtu/+* mice presented with reduced viability, fully penetrant belly spots, tail kinks, and small body size on a predominantly C3H/He genetic background, while *Zma/+* mice on a similar background were observed at the expected frequency and were generally indistinguishable in phenotype from their+/+littermates.

Sequencing of exons 6 and 7 of *Rps7* in 9 inbred strains (A/J, AKR/J, BALB/c, C57BL6/J, C3H/HeJ, CBA, DBA, LP/J, and 101) confirmed that the point mutations detected in *Mtu* and *Zma* are not natural variants. Additionally, we confirmed the presence of the mutations in *Rps7* transcripts using *Mtu*/+ and *Zma*/+ cDNA for sequencing and real-time PCR, respectively. We also demonstrated a lack of complementation between the *Mtu* and *Zma Rps7* alleles by performing an intercross of heterozygous mice from the two lines. Genotyping of newborn offspring revealed that no animals carried both the *Mtu* and *Zma* mutations (N = 27) (Figure S1D). The lack of viable double heterozygotes was consistent with homozygote lethality observed in each line, thus showing non-complementation of the two alleles. Collectively, the similar phenotypes, sequencing data, and genetic analyses provide strong evidence that the mutations identified in *Rps7* are responsible for the observed *Mtu* and *Zma* phenotypes, hereafter referred to as *Rps7^Mtu^* and *Rps7^Zma^*.

### Functional analysis of the RPS7 mutations

The high degree of evolutionary conservation of both mutated amino acids (p.V156G and p.Y177S) suggests that these alterations may disrupt normal function. The consequences of the RPS7^Mtu^ and RPS7^Zma^ mutant proteins were first assessed using the computational analyses PANTHER and SIFT. Results from the PANTHER coding SNP analysis tool [18] suggested that both mutations are likely to be deleterious (PANTHER subPSEC score of −5.00 and −5.06 for RPS7^Mtu^ and RPS7^Zma^, respectively). Similarly, the Sorting Tolerant From Intolerant (SIFT) algorithm [19] predicted both alleles to affect protein function, however, the high degree of conservation in the 72 database sequences at each position resulted in a low confidence level for the prediction (SIFT prediction score 0.00, median conservation 3.59 for both RPS7^Mtu^ and RPS7^Zma^). Collectively, the computational analysis was consistent with the *Rps7^Mtu^* and *Rps7^Zma^* alleles both having functional consequences.

The potential effects of p.V156G and p.Y177S on RPS7 secondary structure (Figure 1D, Figure S2) were also examined. There is no three-dimensional protein structure currently available for metazoan RPS7, therefore we used the available structures from *Tetrahymena thermophila* (PDB ID 2XZM) and *Saccharomyces cerevisiae* (PDB ID 3U5C), whose RPS7 ortholog sequences share 37% and 55% residue identity with mouse RPS7, respectively [20], [21]. RPS7 structural elements appear to be highly conserved across species: the *S. cerevisiae* and *T. thermophila* proteins themselves share only 34% amino acid identity, yet their solved three-dimensional structures superimpose well, and are consistent with secondary structural predictions of mouse RPS7 generated using PSSpred (http://zhanglab.ccmb.med.umich.edu/PSSpred) and PROFsec [22] (Figure S2B). Furthermore, the *S. cerevisiae* and *T. thermophila* sequences each contain a Tyr residue homologous to mouse p.Y177 (Figure S2A), and a Val residue homologous to mouse p.V156 is present in *S. cerevisiae* and substituted conservatively with Ile in *T. thermophila*. The introduction of either p.V156G or p.Y177S into the mouse sequence did not alter predictions of the beta strand and alpha helix at the respective locations of the substitutions (Figure S2B), suggesting that grossly altered secondary structure may not be responsible for the functional consequences of RPS7^Mtu^ or RPS7^Zma^.

**Figure 2:**
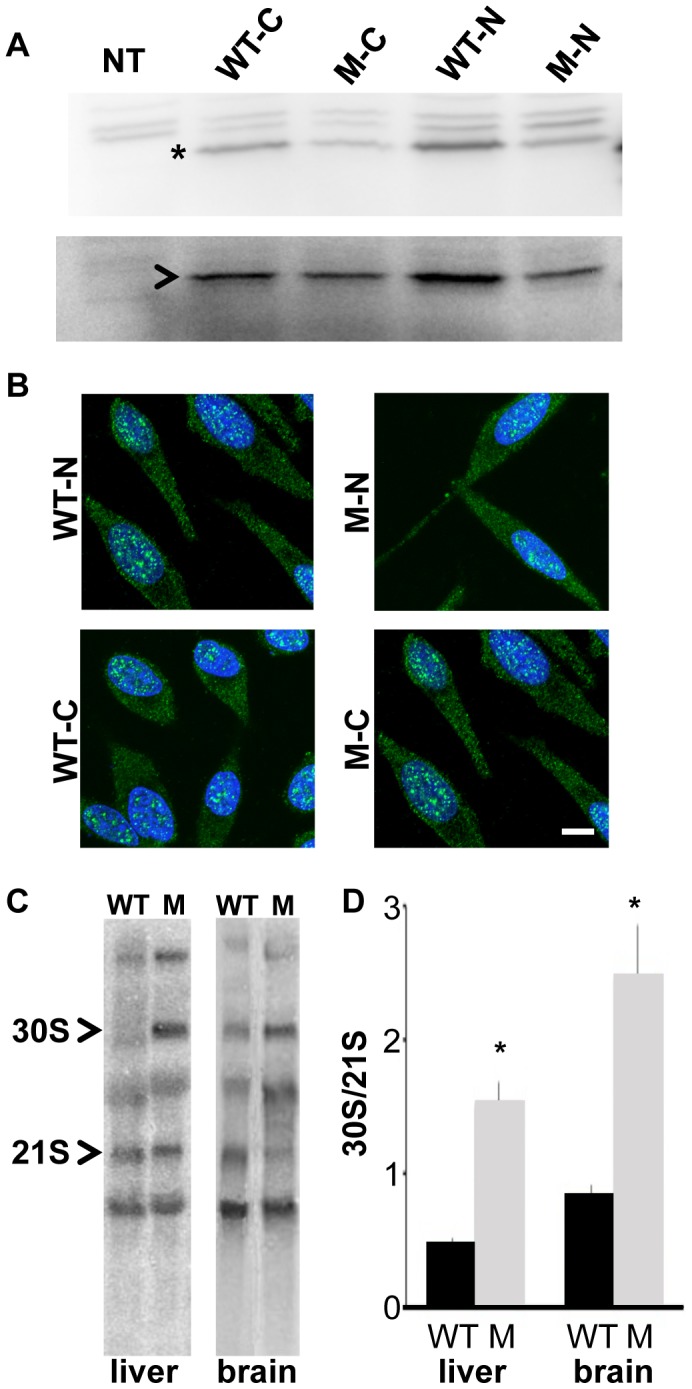
*Rps7^Mtu^* shows reduced function in ribosomal precursor processing. (A) Western blot showing similar levels of expression for N- and C-terminal FLAG-tagged wild-type RPS7 (WT-N and WT-C, respectively) and RPS7^Mtu^ (M-N and M-C, respectively) proteins in HEK-293 cells. The RPS7-specific band is indicated by *, and NPT2 (arrowhead) expression is shown as a control. (B) Subcellular localization of N- and C-terminal FLAG-tagged RPS7 proteins. Wild-type RPS7 and RPS7^Mtu^ both localize to speckles in the nucleus and are observed throughout the cytoplasm. Scale bar = 10 um. All panels are at the same magnification. (C) Representative Northern blot analysis of liver and brain RNA from wild-type (WT) and *Rps7^Mtu^/+* (M) mice detecting various rRNA precursors using a probe within the internal transcribed spacer (ITS1). The 30S and 21S rRNA precursors are indicated. (D) Quantitation of the signals of Northern experiments reported as the ratio between 30S and 21S rRNA precursors was significantly different between *Rps7*+/+ and *Rps7^Mtu^/+* (* indicates p<0.01). The average of the values is reported in the bar graphs with S.E.M.

We next used biochemical analyses to examine the effects of p.V156G and p.Y177S on stability and subcellular localization of the 22 kiloDalton (kDa) protein as well as ribosome assembly and biogenesis. Previous studies have shown that nonsense mutations in RPS19 can result in decreased protein levels, and missense mutations can alter the capacity of RPS19 to localize to the nucleolus [23]. C- and N-terminal FLAG-tagged wild-type, RPS7^Mtu^ and RPS7^Zma^ proteins were transiently expressed in human embryonic kidney (HEK)-293 cells. Western blot analysis revealed no differences in protein levels for any of the RPS7 mutant proteins (Figure 2A, Figure S3A). The remaining *in vitro* studies were focused on the potentially more severe RPS7^Mtu^ mutant protein. Analysis of the FLAG-tagged proteins revealed no notable differences in subcellular localization of the RPS7^Mtu^ protein (Figure 2B). To verify the capacity of RPS7^Mtu^ to be incorporated into the ribosome, cytoplasmic extracts from transiently transfected HEK-293 cells were fractionated to separate ribosomes and ribosomal subunits (in the pellet) from free cytoplasmic proteins (in the supernatant). A similar fraction of all transfected RPS7 proteins was observed in the ribosomal pellet (Figure S3B) indicating that the mutation does not alter RPS7 assembly into the ribosome. To confirm this finding, cytoplasmic extracts from *Rps7^Mtu^*/+ liver were analyzed by ultracentrifugation through sucrose gradients, and the ratio between the peaks of the ribosomal subunits in the absorbance profile was determined. This ratio can reveal defects in the synthesis of one of the two subunits, such as the net increase of the 60S/40S ratio seen in cultured human cells following depletion of single RPs [24], [25]. However, the observed 60S/40S ratio in *Rps7^Mtu^*/+ liver was similar to control (Figure S3C), indicating that RPS7^Mtu^ does not drastically alter assembly of the ribosomal subunits.

We further assessed whether RPS7^Mtu^ affects ribosomal biogenesis by analyzing the pre-rRNA maturation pattern. Alterations are visualized by the accumulation of specific rRNA precursors: a defect in large subunit RPs affects cleavage of 28S precursors, whereas a defect in small subunit RPs alters that of 18S precursors. Northern blot analysis of total RNA from wild-type and *Rps7^Mtu^*/+ tissue, using a probe for the internal transcribed spacer (ITS) 1 of pre-rRNA that anneals to 18S rRNA precursors [26], [27], indicated that 18S rRNA pre-rRNA processing was altered both in liver and brain from *Rps7^Mtu^*/+ mutant mice (Figure 2C). Quantitative measurement of the hybridization signals showed a significant accumulation of the 30S precursor in *Rps7^Mtu^*/+ (indicated by an increased 30S/21S ratio; Figure 2D), confirming altered rRNA precursor processing and demonstrating that the RPS7^Mtu^ mutation has functional consequences on ribosomal biogenesis.

### Characterization of the *Rps7* phenotype

#### Skeletal and eye malformation

**Figure 3:**
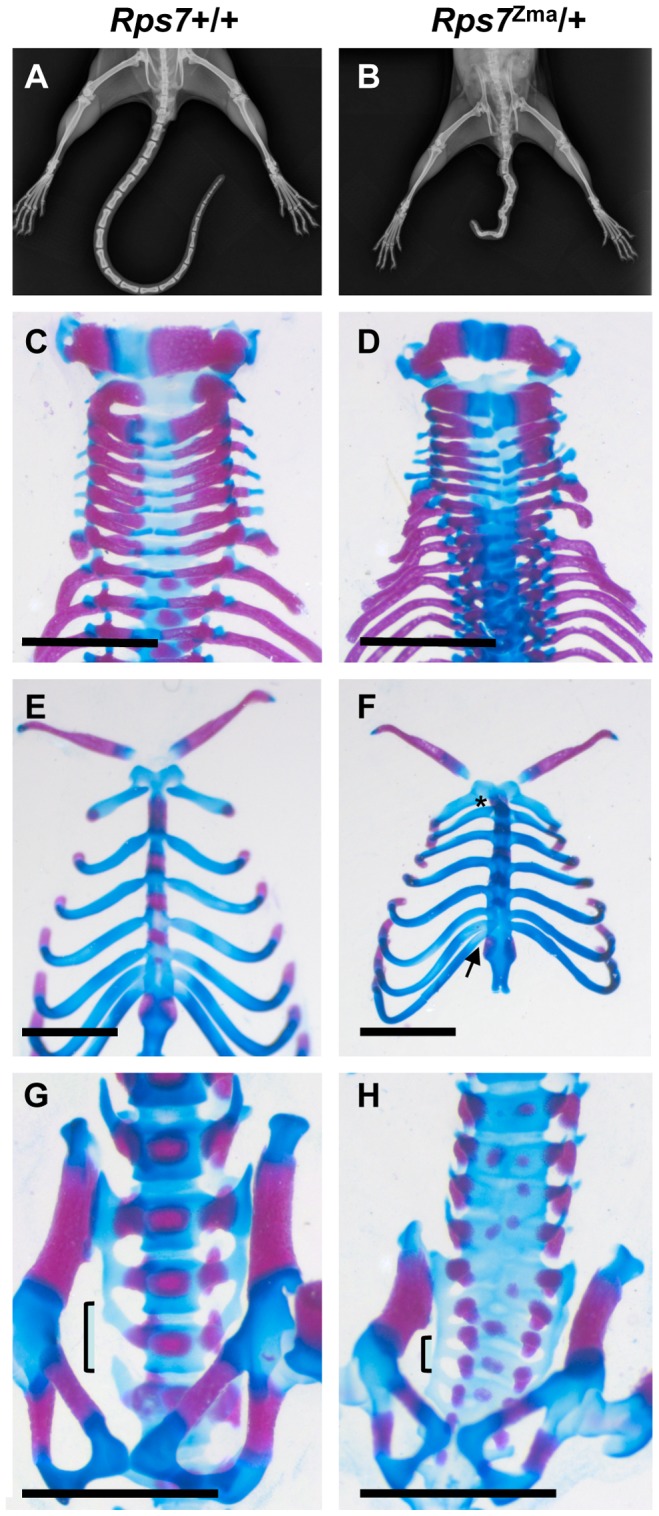
Skeletal abnormalities in *Rps7* mutants. (A, B) Comparative X-rays of adult *Rps7*+/+ (A) and *Rps7^Zma^/+* (B) mice show severe vertebral fusion leading to tail kinking in an *Rps7^Zma^/+* mutant. (C–H) Alcian blue and alizarin red skeletal staining of late gestation *Rps7*+/+ (C, E, G) and *Rps7^Zma^/+* (D, F, H) embryos. (C, D) Disorganization within the neural arches of *Rps7^Zma^/+* is evident in a dorsal view of the cervical vertebrae. (E, F) Asymmetric attachment of 8 ribs to the sternum (arrow) and a shortened first sternebrae (*) are indicated in *Rps7^Zma^/+*. (G, H) Delayed development and disorganization of the ossification centers is apparent in the lumbar and sacral vertebrae. The sacral vertebrae show abnormal fusing of additional vertebrae (bracketed region). Scale bars: C,D,G,H = 2 mm; E,F = 200 µm.

X-ray examination of adult *Rps7^Zma^*/+ mice showed that vertebral fusion is responsible for the tail kinks (Figure 3A, 3B). Similar results were observed for *Rps7^Mtu^*/+ adult mice, indicating that in both mutants, defective somitogenesis occurs as a result of RPS7 alterations. Decreased vertebrae numbers were also observed in both mutants; a reduction in total vertebrae number in *Rps7^Mtu^*/+ (52–55 versus 55–57 in wild-type) and in caudal vertebrae number in *Rps7^Zma^* ^/+^ (16–19 versus 20–22 in wild-type). The decreased vertebrae numbers indicate defective tail bud function associated with defects in RPS7. Alcian blue and alizarin red staining of late gestation *Rps7*+/+ and *Rps7^Zma^*/+ embryos (N = 3 for each genotype) revealed further *Rps7^Zma^*/+-associated skeletal abnormalities, including general disorganization of vertebral processes and arches in the cervical vertebrae (Figure 3C, 3D). The anterior tuberculum was appropriately located on C6, however a very small partial rib was observed on C7 (in 2 *Rps7^Zma^*/+ embryos), indicating an incompletely penetrant transformation of C7 to T1. Within the thoracic region, we observed a shortened first sternebrae and asymmetric attachment of 8 ribs to the sternum rather than the usual 7 (in all 3 *Rps7^Zma^*/+ embryos) (Figure 3E, 3F). Within the lumbar and sacral regions, a severe developmental delay was observed, and disorganization of the ossification centers was evident with some ossification centers being duplicated (Figure 3G, 3H). Additionally, we observed a transformation of L1 to T13 (in all 3 *Rps7^Zma^*/+ embryos) and also asymmetric fusion of the transverse processes of the first 5–6 sacral vertebrae (in 2 *Rps7^Zma^*/+ embryos) rather than the 3–4 observed in *Rps7*+/+ (Figure 3H). Collectively the skeletal phenotypes observed in *Rps7* mutants are consistent with an anterior transformation of the axial skeleton.

**Figure 4:**
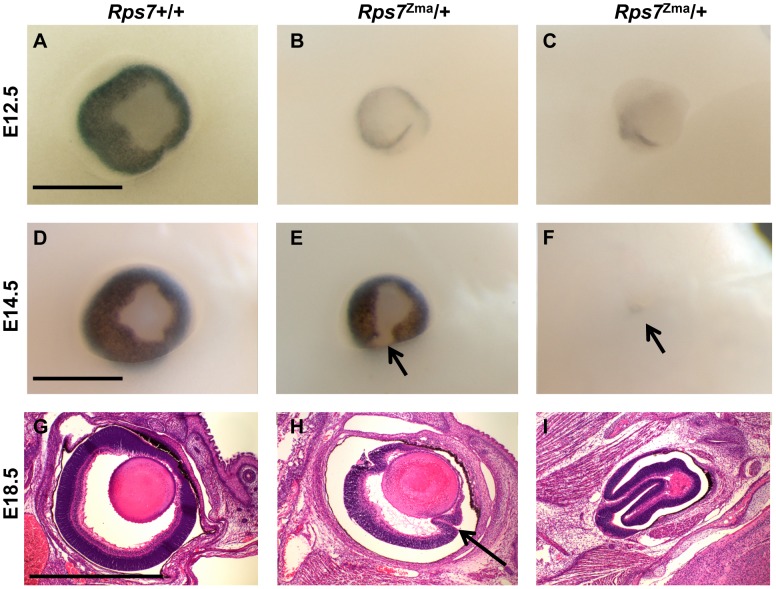
Eye dysmorphology in *Rps7* mutants. Representative whole-mount images from one *Rps7*+/+ and two different *Rps7^Zma^/+* embryos are shown at E12.5 (A–C) and 14.5 (D–F). In addition, H&E stained sagittal sections through the eye are shown for one *Rps7*+/+ and two different *Rps7^Zma/^+* embryos at E18.5 (G–I). The eye dysmorphology of *Rps7^Zma^/+*mutants ranges in severity from minor unilateral or bilateral uveal coloboma (E, H) to severe microphthalmia resulting in disorganized eye structures (C, F, I). Arrows in E and F mark examples of coloboma and extreme microphthalmia, respectively. Arrow in H marks abnormal folding of the retinal layers. All images are oriented with anterior up, rostral to the right. Within each age group/row, all genotypes are shown at the same magnification. Scale bars = 0.5 mm.

Frequent microphthalmia and uveal coloboma were also observed as features of the *Rps7* mutant phenotype (Figure 4). *Rps7^Zma^*/+ embryos showed eye malformations ranging from minor delay in optic fissure closure to severe microphthalmia and internalization of the entire eye structure. Whole mount microscopic imaging of E12.5 embryos revealed that 100% of *Rps7^Zma^*/+ embryos exhibited a unilateral or bilateral delay in closure of the optic fissure, as compared to *Rps7*+/+ embryos which all showed normal optic fissures (N = 5 *Rps7^Zma^*/+, N = 7 *Rps7*+/+) (Figure 4A–4C). By E14.5, a time when wild-type embryos have complete fissure, 100% of *Rps7^Zma^*/+ embryos still displayed optic fissure closure defects either unilaterally (N = 2) or bilaterally (N = 3) (Figure 4D–4F). Additionally, more severe microphthalmia and internalization of the eye was observed in 20% of *Rps7^Zma^*/+ embryos (Figure 4F, 4I; 1 out of 5 at both E12.5 and E14.5).

#### Circulating blood chemistry and erythroid differentiation

**Figure 5:**
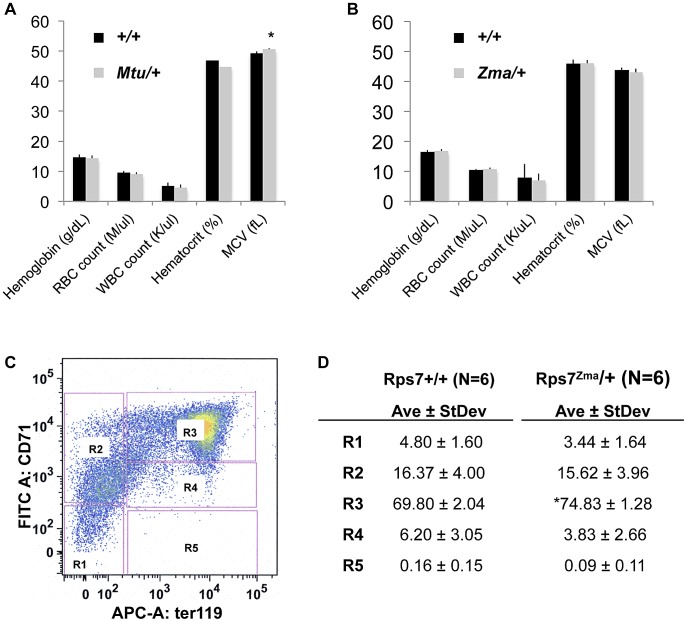
Peripheral-blood parameters appear normal with slight developmental delay in *Rps7^Zma^/+* fetal liver red cell precursors. (A) *Rps7^Mtu^/+* mice displayed similar complete blood count (CBC) values to their wild-type littermates with the exception of a very slightly elevated mean corpuscular volume (MCV) (* indicates p<0.05; N = 7). (B) *Rps7^Zma^/+* mice did not differ significantly from their wild-type littermates in any CBC measurements (N = 5). (C) A typical example of FACS analysis of fetal liver cells. The 5 characterized erythroid cell populations, R1–R5, are boxed in pink. (D) The average values and standard deviation for each fetal liver erythroid cell population are given as a percentage of total viable cells in Rps7+/+ and *Rps7^Zma^/+* samples (* indicates p<0.001; N = 6).

Because human *RPS7* mutations are associated with DBA [11], a disease characterized by red cell aplasia, we undertook an initial characterization of circulating blood in adult *Rps7^Mtu^*/+ mice (on a C3H/He background) and *Rps7^Zma^*/+ mice (on a mixed C57BL/6J; BALB/cJ background). Neither *Rps7* mutant allele caused any significant differences from *Rps7*+/+ littermates in total hemoglobin, red blood cell (RBC) counts, white blood cell (WBC) counts, or hematocrit (Figure 5A, 5B, Table S1). Mean corpuscular volume (MCV) was slightly elevated in *Rps7^Mtu^/+* mice (elevated 1.026 fold over wild-type, p<0.05) (Figure 5A), however no significant difference in MCV was observed in *Rps7^Zma^/+* mice (Figure 5B). Additional analyses of plasma biochemistry revealed no significant differences between *Rps7^Mtu^/+* and *Rps7*+*/+* animals in major electrolytes and metabolic indicators (Na^+^, K^+^, Cl^−^, urea, creatinine, ALP, AST, ALT, total cholesterol, triglycerides, iron, amylase, and free fatty acids) (Table S2).

To further assess erythroid development of *Rps7^Zma^/+* mice, which exhibit perinatal lethality on a C57BL/6J background, E13.5 *Rps7^Zma^/+* fetal liver samples were characterized by flow cytometry. We simultaneously used monoclonal antibodies against the erythroid cell lineage markers CD71, expressed at higher levels during early stages of erythroid differentiation, and Ter119, expressed at higher levels as erythrocytes mature, to profile 5 characteristic populations of erythroid precursors (R1 to R5) [28], [29] (Figure 5C). These cell populations correspond to increasingly differentiated developmental stages, with R1 marking erythroid progenitors and proerythroblasts, R2 marking proerythroblasts and early basophilic erythroblasts, R3 marking all basophilic erythroblasts, R4 marking chromatophilic and orthochromatophilic erythroblasts, and R5 marking late orthochromatophilic erythroblasts and reticulocytes. Compared to their *Rps7*+/+ littermates, *Rps7^Zma^*/+ E13.5 embryos showed a significant increase in the percentage of R3 cells (p<0.001) suggesting that erythroid precursors could be blocked in their maturation (Figure 5D). However, analysis of erythroid cell populations of E14.5 fetal livers showed that the percentages of cells in the R2–R5 populations were indistinguishable between *Rps7*+/+ and *Rps7^Zma^*/+ genotypes (Table S3). The presence of the more mature R4 and R5 cells at E14.5 indicates erythroid differentiation progresses, rather than being blocked at R3. Alternatively, the increase in R3 cells at E13.5 may result from a general developmental delay in *Rps7^Zma^*/+ embryos. This was confirmed by inspection of gross anatomical features of crown-rump length, along with eye, limb, and craniofacial development at E11.5–13.5, which showed that *Rps7^Zma^*/+ embryonic development is delayed by approximately one day relative to that of *Rps7*+/+ littermates (Figure S4). While we cannot rule out subtle alterations in erythroid maturation that may cause the differences observed in the precursor populations, these analyses collectively suggest that these two mutant alleles of *Rps7* do not significantly impair red blood cell production or differentiation.

#### Early effect on melanoblast development

**Figure 6:**
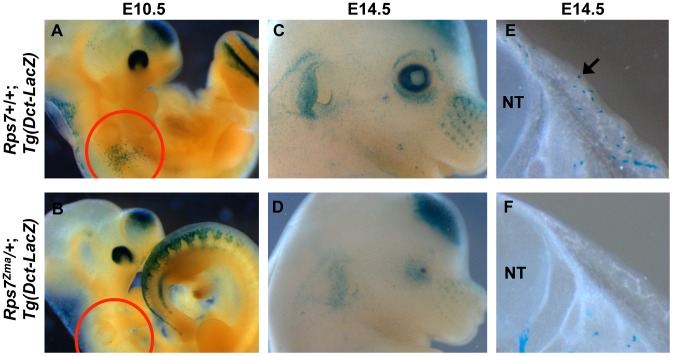
Melanoblast numbers are reduced in *Rps7^Zma^/+* mutants. (A, B) In transgenic embryos carrying the melanoblast reporter *Tg(Dct-LacZ)*, whole mount staining showed that Dct-positive melanoblasts are significantly reduced at E10.5 in *Rps7^Zma^/+*; *Tg(Dct-LacZ)* mice (A) compared to *Rps7*+/+;*Tg(Dct-LacZ)* littermates (B). The reduction is noticeably apparent over the otic region (red circle). (C,D) Whole mount staining of E14.5 *Rps7^Zma^/+*; *Tg(Dct-LacZ)* embryos showed that these embryos (D) also display a reduction in melanoblasts relative to *Rps7*+/+;*Tg(Dct-LacZ)* littermates (C). The microphthalmia observed in *Rps7^Zma^/+* mice is apparent in (D). (E, F) Consistent with the whole-mount observations, transverse vibratome sections through the trunk of E14.5 embryos revealed very few melanoblasts in the developing skin of *Rps7^Zma^/+*; *Tg(Dct-LacZ)* mice (F) as compared to the numerous melanoblasts seen in *Rps7*+/+;*Tg(Dct-LacZ)* littermates (arrow, E). Blue punctate staining indicates positive signal in melanoblasts. In all pairs of images, *Rps7*+/+ and *Rps7^Zma^/+* are at the same magnification. NT = neural tube.

To assess the effects of RPS7 mutation on melanocyte development, we characterized *Rps7^Zma^/+* mutant embryos from E10.5 to 14.5. To mark developing melanoblasts, we generated transgenic *Rps7^Zma^/+* embryos carrying the melanoblast reporter *Tg(Dct-LacZ)* [30]. In these *Rps7^Zma^/+*; *Tg(Dct-LacZ)* embryos, *Dct*-positive melanoblasts were notably reduced by E10.5 relative to *Rps7*+/+; *Tg(Dct-LacZ)* littermates (Figure 6A, 6B). At E14.5, a significant reduction in melanoblast number was still observed in both the head and trunk of *Rps7^Zma^/+*; *Tg(Dct-LacZ)* mice compared to *Rps7*+/+; *Tg(Dct-LacZ)* littermates (Figure 6C–6F). Mutation of *Rps7* did not cause a complete loss of melanoblasts, however, as *Dct-*positive cells could still be observed at E14.5 in regions where melanoblast density is normally highest, particularly around the developing pinna and eye (Figure 6C, 6D). This severe melanoblast reduction in *Rps7^Zma^/+* embryos was independently confirmed by *in situ* hybridization with a probe targeting mRNA for the melanoblast/melanosome-specific protein *Pmel17* (Figure S5). Qualitative observation of *Pmel17-*expressing melanoblasts showed that E12.5 *Rps7^Zma^/+* mutant embryos displayed a marked reduction in melanoblasts relative to E12.5 *Rps7*+/+ littermates. The reduction in melanoblasts is more severe than can be attributed to the developmental delay observed in *Rps7^Zma^/+* mutants, as E12.5 *Rps7^Zma^/+* embryos showed a reduced melanoblast number even in comparison to E11.5 *Rps7*+/+ embryos (Figure S5). A combination of *Rps7* and *Sox10* haploinsufficiency (*Rps7^Zma^/+; Sox10^LacZ^/+* double heterozygotes) further reduced melanoblast number at E12.5 (Figure S6), consistent with the increased spotting observed in rare *Rps7^Zma^/+; Sox10^LacZ^/+* pups that survived postnatally (Figure S7A, S7B).

Postnatally, we observed that melanocytes were present in the tail skin of *Rps7^Zma^/+* mice (Figure S7E, S7F), yet no dark skin phenotype occurred in the tails or footpads of adult *Rps7^Zma^/+* or *Rps7^Mtu^/+*mice (Figure S7C–S7F). This indicates that these *Rps7* mutations do not cause epidermal melanocytosis, as was previously observed for mutation of *Rps19* and *Rps20* [5]. Collectively, these data show that mutation of *Rps7* causes a severe, early reduction in melanoblasts that is sufficient to account for the white belly spotting observed in viable heterozygotes.

#### Enlarged ventricles, cortical thinning, and deficits in working memory

**Figure 7:**
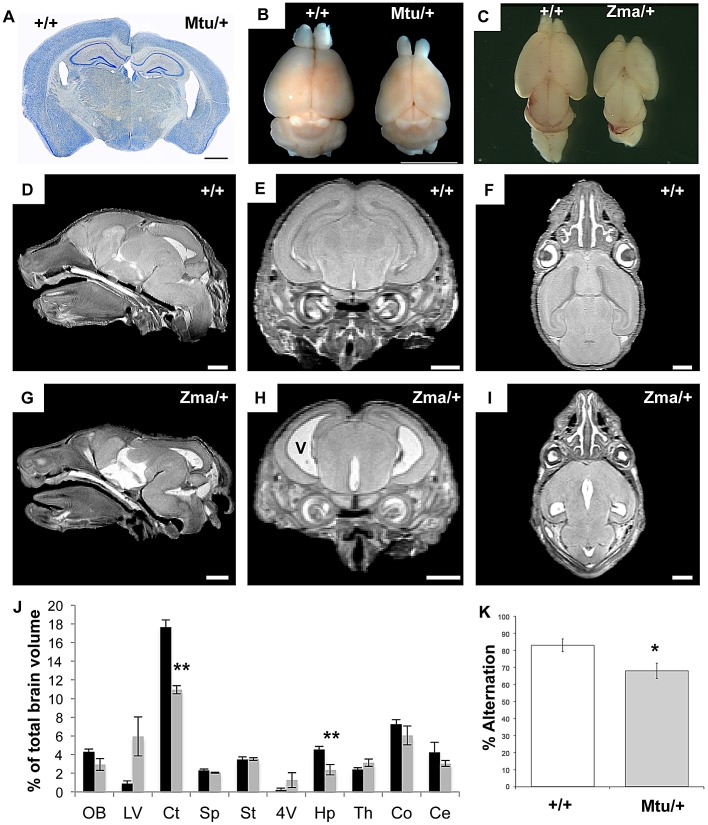
Brain size and behavioral abnormalities in *Rps7* mutants. (A) A Nissl stained coronal section of a 5 month old *Rps7^Mtu^/+*brain shows a thinner cortex and larger ventricles when compared to an *Rps7*+/+ littermate. (B, C) Dissected whole brains show that the cortex is reduced in size in adult *Rps7^Mtu^/+* (B) and postnatal day 0 *Rps7^Zma^/+*mice (C) when compared to *Rps7*+/+ littermates. (D–I) Magnetic Resonance Microscopy (MRM) was used to visualize brain development in late gestation (E18.5) *Rps7*+/+(D–F) and *Rps7^Zma^/+*(G–I) embryos. Enlarged ventricles (v in panel H) were apparent in all *Rps7^Zma^/+* samples (N = 3). Representative slices are shown in sagittal (D,G), coronal (E,H), and axial (F,I) views. Scale bars = 1 mm. (J) The volume of each brain region was quantitated as a percentage of total brain volume in *Rps7*+/+(black columns) and *Rps7^Zma^/+*(gray columns) (N = 3). Abbreviations: Olfactory bulbs (OB), lateral ventricles (LV), cortex (Ct), septum (Sp), striatum (St), 4^th^ ventricle (4V), hippocampus (Hp), thalamus (Th), colliculi (Co), cerebellum (Ce). ** indicates p<0.005. (K) Assessment of working memory by measuring spontaneous alternation in a T-maze showed a significant difference between *Rps7^Mtu^/+*mice and *Rps7*+/+ littermate controls (* indicates P = 0.01, N = 9). In all panels +/+ = *Rps7*+/+; Mtu/+ = *Rps7^Mtu^/+*; Zma/+ = *Rps7^Zma^/+*.

We examined the neuroanatomical features of the *Rps7* mutants, employing histological techniques and magnetic resonance microscopy (MRM). Gross observations and histological staining revealed marked cortical thinning accompanied by enlarged ventricles in adult *Rps7^Mtu^/+* (Figure 7A–7B) and perinatal *Rps7^Zma^/+* mice (Figure 7C, and data not shown). To investigate this phenotype further, we undertook MRM imaging of *Rps7^Zma^/+* mice at E18.5 (N = 3) (Figure 7D–7I). Following manual segmentation of 10 major anatomic structures, the volume of each brain region was calculated as a percentage of the total brain volume (Figure 7J) and 3-dimensional (3D) rendering with smoothing was performed to generate 3D representations (Video S1 and Video S2). This quantitative analysis showed that the *Rps7^Zma^/+* CNS phenotypes originate during development and confirmed a significant reduction in cortical (P = 0.0002) and hippocampal (P = 0.0044) size in *Rps7^Zma^/+* mutants. Although lateral ventricle volume was not significantly different between these small groups, qualitative observations of ventricle size at dissection, in MRM images, and in histological sections consistently indicated *Rps7^Zma^/+* mutants had enlarged ventricles, leading us to conclude that enlarged ventricles are a consistent feature of the *Rps7* phenotype.

We investigated whether these gross neuroanatomical phenotypes affected the behavior of the *Rps7^Mtu^/+* mice, assessing locomotor activity, anxiety and working memory. The results showed that while *Rps7^Mtu^/+* mutants exhibited normal locomotor activity and anxiety-related phenotypes, they displayed deficits in working memory. *Rps7^Mtu^/+* mutants displayed similar behavior to *Rps7*+/+ controls in an open-field test, with no significant differences in total distance travelled in the open field or time spent in the center (Figure S8A). Similarly, there were no significant differences between *Rps7*+/+ controls and *Rps7^Mtu^/+*littermates when assessed on the elevated plus-maze (Figure S8B–S8E), nor did *Rps7^Mtu^/+* mutant animals display any ataxia that would be indicative of motor dysfunction. We also investigated whether *Rps7^Mtu^/+* mice displayed any defects in working memory using a spontaneous alternation test, which can indicate hippocampal dysfunction [31]. The mouse is placed in the start arm of an enclosed T-maze (the base of the “T”), and allowed to enter a goal arm (the two arms of the top of the “T”) of its own choice. Upon repeating this test, the mouse can demonstrate memory of its previous choice by entering the arm not visited before, a behavior called spontaneous alternation. Here we observed a significant difference between *Rps7*+/+ controls, which exhibited an average alternation frequency of 83%, and *Rps7^Mtu^/+* mutants, which exhibited an average alternation frequency of only 68% (N = 9, P = 0.01) (Figure 7K). Collectively, the MRM and behavioral data indicate that *Rps7* is required for normal development of the central nervous system, particularly the telencephalon, and that mutations in this gene have the capacity to affect hippocampal-dependent behavior.

#### Increased neuronal apoptosis in *Rps7* mutants

**Figure 8:**
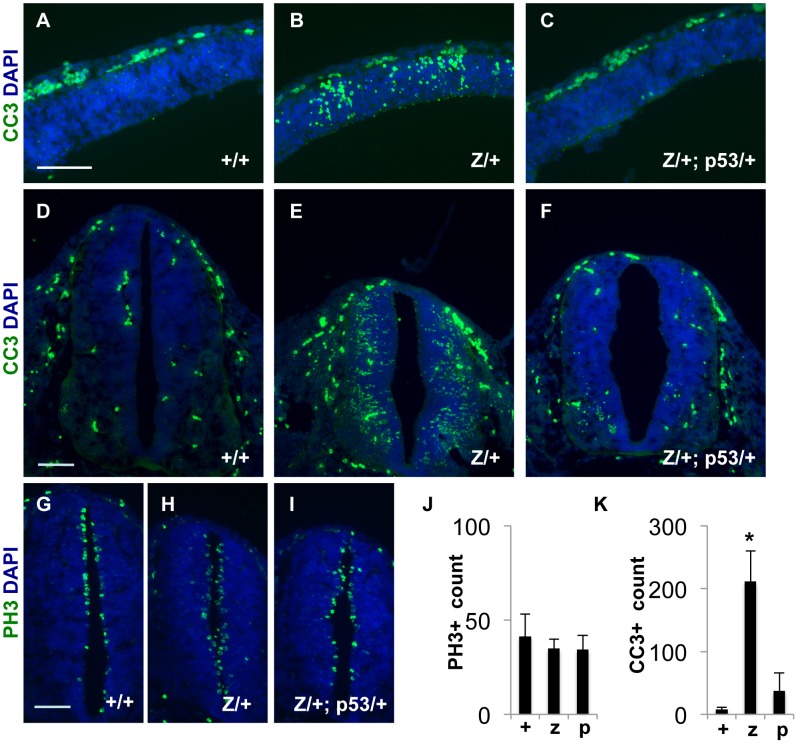
Increased apoptosis occurs in the developing CNS of *Rps7^Zma^/+* mutant embryos; however, this apoptosis is reduced in *Rps7^Zma^/+*; *Trp53^KO^/+* embryos. (A–C) Increased apoptosis was observed in E11.5 *Rps7^Zma^/+* (Z/+) coronal sections through the neocortex compared to *Rps7*+/+ (+/+) and *Rps7^Zma^/+*; *Trp53^KO^/+* (Z/+;p53/+), as measured by cleaved caspase-3 (CC3) staining shown in green. (D–F) Apoptosis was also relatively increased in neural tube cross-sections of *Rps7^Zma^/+* embryos at E11.5 (E). (G–I) Cellular disorganization was apparent in mitotic, phospho-histone H3-positive (PH3+) cells surrounding the lumen of the neural tube in E11.5 *Rps7^Zma^/+* embryos (H). (J) E11.5 *Rps7^Zma^/+* (z) embryos showed no difference from *Rps7*+/+(+) or *Rps7^Zma^/+*; *Trp53^KO^/+*(p) in total counts of PH3+ cells surrounding the lumen of the neural tube. (K) CC3+ cell counts confirmed significantly increased apoptosis in E11.5 *Rps7^Zma^/+* (z) neural tube as compared to *Rps7*+/+ (+) or *Rps7^Zma^/+*; *Trp53^KO^/+* (p) (* indicates p<0.001). Scale bars: in A, D, G = 100 µM with equivalent magnification across all genotypes.

Our histological studies also revealed that late gestation *Rps7^Zma^/+* embryos exhibited pyknotic nuclei in the white matter tracts of the spinal cord (Figure S9), consistent with CNS degeneration. As mutations in other RPs have been associated with increased cellular apoptosis [32]–[37], we used immunohistochemistry to detect cleaved caspase-3 (CC3) within the CNS. When compared to normal littermates, *Rps7^Zma^/+* embryos showed increased apoptosis in the cortex and neural tube at both E11.5 (Figure 8A, 8B, 8D, 8E) and E12.5 (data not shown). Although mitotic, phospho-histone H3-positive (PH3+) cells surrounding the lumen of the neural tube appeared slightly disorganized in E11.5 and E12.5 *Rps7^Zma^/+*embryos (Figure 8G, 8H and data not shown), cell counting revealed no significant difference in PH3+ cell number per section compared to *Rps7*+/+ littermates (Figure 8J). In contrast, CC3 counts confirmed a significant increase in apoptosis in the neural tube of E11.5 *Rps7^Zma^/+* embryos compared to *Rps7*+/+ (N = 3, p<0.001) (Figure 8K). Consistent with the location of apoptosis and with a putative role for RPS7 in CNS development, *in situ* hybridization studies on *Rps7*+/+ embryos revealed that *Rps7* is highly expressed in proliferative regions, including the ventricular zone (Figure S10). As TRP53 levels are altered in other ribosomal mutants, we also investigated the expression of TRP53 in the developing CNS of *Rps7* mutants. Using immunohistochemistry, we observed an increase in TRP53 staining in the cortex (E11.5) and neural tube (E11.5 and E12.5) of *Rps7* mutants consistent with increased TRP53-mediated apoptosis (Figure S11). Collectively, the histology and immunohistochemistry data confirm that massive cell death in the *Rps7* mutant CNS occurs at the onset of neurogenesis.

#### Phenotypic suppression of *Rps7* mutant phenotypes by*Trp53* haploinsufficiency

In an attempt to restore heterozygote viability by suppressing apoptosis, we crossed the *Rps7^Zma^* mutation onto a *Trp53* mutant background. *Trp53* mutations have been used to successfully suppress phenotypes in other RP mutants [5], [35]. *Rps7^Zma^* mice were originally identified on a mixed genetic background, and heterozygote lethality in *Rps7^Zma^/+* mice increased as the mutation was outcrossed onto a C57BL/6J background. Thus we used early generation, mixed background *Rps7^Zma^/+* mice to establish a cross with *Trp53* mutant mice on a C57Bl/6J background (B6.129S2-Trp53^tm1Tyj/J^, hereafter *Trp53^KO^*). Within a few generations of outcrossing, no viable *Rps7^Zma^/+* mice were observed after birth, as expected. However, *Rps7^Zma^/+; Trp53 ^KO^/+* double heterozygotes were recovered at the expected 50% frequency, suggesting restoration of *Rps7^Zma^/+* viability in the context of *Trp53* haploinsufficiency (Table S4). In addition to restoring viability, *Trp53* mutation fully suppressed the *Rps7^Zma^-*associated vertebral fusion phenotype that was observed in earlier generations of *Rps7^Zma^/+* mice (no tail kinks observed in >25 *Rps7^Zma^/+; Trp53^KO^/+* mice). In most cases *Rps7^Zma^/+; Trp53^KO^/+* double heterozygotes were indistinguishable from their wild-type littermates after weaning, with the exception of a very small belly spot observed in 47% of *Rps7^Zma^/+; Trp53^KO^/+* mice. *Trp53* haploinsufficiency also suppressed the phenotypes of gross embryonic developmental delay (Figure S4), the subtle delay in erythroid maturation (Table S3), and CNS apoptosis (Figure 8C, 8F, 8K). In summary, the pleiotropic phenotypes observed in *Rps7^Zma^/+* mice all appeared to be suppressed by *Trp53* haploinsufficiency.

## Discussion

In this paper we present genetic, functional, and phenotypic evidence that the montu (*Mtu*) and zuma (*Zma*) mouse lines isolated from independent ENU screens harbor distinct point mutations in *Rps7*. These first-reported alleles of *Rps7* in mice cause similar phenotypes including small body size, tail abnormalities, mid-ventral white spotting, eye defects, and an underdeveloped cerebral cortex.

We provide functional evidence that the *Rps7^Mtu^* mutation (p.V156G) affects ribosome biogenesis. The altered *Rps7^Mtu^* 30S/21S ratio is consistent with the altered pre-rRNA maturation reported for a DBA patient harboring an *RPS7* donor splice-site mutation (c.147+1G>A) that results in accumulation of 30S and 45S precursors [11]. A requirement for RPS7 during rRNA maturation is supported by other studies showing a role for human RPS7 in early stages of 45S rRNA processing [24] or the nuclear stages of 40S maturation [38]. In yeast, depletion of the two RPS7 paralogs (S7a and S7b) results in a severe growth defect [39], [40] and S7 has been hypothesized to play an early role as a component of the small subunit processome [41] as well as a later role in ribosome biogenesis during assembly of polysomes [42]. However, the mechanism underlying the altered rRNA maturation in these murine *Rps7* mutants is not clear, as neither the *Rps7^Mtu^* nor the *Rps7^Zma^* mutation is predicted to grossly disrupt protein secondary structure, and RPS7^Mtu^ protein is correctly localized and incorporated into ribosomes in cultured cells. Presumably, the mutant RPS7 proteins could be altered in their interactions with rRNA, other ribosomal proteins, or non-ribosomal proteins. The possibility of interaction with non-ribosomal proteins is intriguing given the position of RPS7 at the surface of the ribosome (Figure S2C, S2D) in close proximity to the binding site for eIF4G [21], a eukaryotic initiation factor important for assembling the pre-initiation complex. Interestingly, most other known, viable mouse RP mutations also occur in eukaryotic-specific RPs located at the surface of the ribosome. Additional work is needed to determine the precise role RPS7 plays in mammalian ribosome biogenesis and how the *Rps7^Mtu^* and *Rps7^Zma^* mutations disrupt this function.

While modeling the pre-rRNA processing defect of an RPS7 DBA patient, these *Rps7* mutant mice do not replicate the characteristic DBA phenotype of severe anemia. This lack of an anemia phenotype is not entirely unexpected, since the analysis of many mouse mutants suggests that mice as a species are generally less sensitive than humans to any gene haploinsufficiency [43], [44]. This is exemplified by the fact that, despite frequent mutation of RPS19 in DBA, *Rps19* heterozygote knockout mice have been described with normal hematopoiesis [34], [45] and an ENU allele of *Rps19* displays only a mild erythrocyte phenotype, along with an elevated MCV that parallels the slightly elevated MCV in *Rps7^Mtu^* [5]. It is possible that further studies of *Rps7* alleles on different genetic backgrounds or in mice with more severe alleles may reveal additional red blood cell phenotypes. In the future, comparison among mouse DBA models may provide insight into why the murine and human hematological phenotypes do not fully overlap, and may also reveal why mutation in RPS19 has a uniquely high frequency in DBA compared to other RPs that cause the disease.

Both *Rps7* mutants display a variety of skeletal phenotypes that collectively suggest that normal ribosome biogenesis is required for three distinct stages of somite development, which governs subsequent axial skeleton formation. First, the decreased number of tail vertebrae indicates an insufficient production of somite progenitors. Second, the mutant vertebral fusion leading to tail kinks is indicative of incorrect somite border formation. Third, the mutants exhibit an array of axial skeleton defects consistent with an anterior transformation, indicating that somite identity is perturbed. Widespread disturbances of the skeletal system encompassing each of these three defects are also found in *Rpl38* (tail short, *Ts*) [46], [47] and *Rpl24* (belly spot and tail, *Bst*) mutants [6], [48]. Indeed, there are striking similarities in the defects observed in *Rpl38* and *Rps7* mutants, including specific transformations within the cervical (C7 to T1), thoracic (T8 to T7), lumbar (L1 to T13) and sacral (fusion of additional transverse processes) regions. Mutations in *Rpl38* and *Rpl24* cause additional disturbances of the appendicular skeleton not observed in *Rps7*, however, given the similarities of these mutants to *Rps7* it will be interesting to see if appendicular skeletal defects are observed with different *Rps7* alleles and/or genetic backgrounds. Interestingly, severe skeletal malformations have not been reported for five other murine RP mutants (*Rps19*, *Rps20, Rpl22*, *Rpl27a*, *Rpl29*) [5], . This suggests that either mutation of RP proteins do not universally affect skeletal development, or skeletal defects remain to be identified in these other RP mutants. In the case of *Rps7*, the severity of skeletal defects was modified by both genetic background and by allele, suggesting that detailed characterization in existing and additional RP mutants is necessary to further explore the role of RP proteins in skeletal development.

We show that *Rps7^Zma^*-mediated white spotting results from a severe developmental reduction in melanoblasts. A developmental reduction in melanoblasts has also been reported in *Rps19* and *Rps20* heterozygous mutants, however, these mutants subsequently develop epidermal melanocytosis [5], a phenotype not seen in either *Rps7* mutant. This difference is unlikely to be due to genetic background, as both *Rps7* mutants and *Rps19* and *Rps20* mutants were examined on a predominantly C3H background. Alternatively, the difference could be a more severe developmental reduction in melanoblast numbers in *Rps7* mutants, resulting in fewer melanoblasts available at later time points to generate epidermal melanocytosis. Another possibility is that loss of *Rps7* may not have the same effect in keratinocytes as loss of *Rps6*, *Rps19* and *Rps20*, which are thought to act in keratinocytes to produce TRP53-mediated dark skin [5]. Our *in vitro* experiments with *Rps7* (Figure 2 and Figure S3) suggest that these mutations are hypomorphic alleles, so a third possibility is that allele severity, rather than gene-specific function, could account for pigmentation phenotype differences among RP mutants.

The mouse *Rps7* mutants display additional developmental defects of reduced cortical and hippocampal size. We hypothesize that cell death within the developing CNS is sufficient to account for the neocortical thinning we observe in both *Rps7* mutant lines. We also hypothesize that apoptosis within the developing telencephalon might account for the deficit in working memory observed in *Rps7^Mtu^/+* heterozygotes. To our knowledge, this is a novel developmental brain abnormality in mouse arising from RP haploinsufficiency, differing substantially from the only other report of neuronal defects in RP mutant mice, that of cerebellar abnormalities and ataxia in *Rpl27a* sooty foot mutants [7]. Interestingly, the neuronal phenotypes in mouse *Rps7* mutants correlate with the report of microcephaly as one of 11 congenital craniofacial anomalies that can be associated with DBA [51], and are consistent with RP mutant phenotypes observed in different model organisms. In zebrafish, gene-specific neuronal phenotypes were seen in a subset of knocked-down RPs [4] and further studies of RPL11 revealed brain deformities and reduced neuronal progenitor cells along with apoptosis in the affected regions [32]. In the zebra finch, RPL17 and RPL37 both regulate the sexually dimorphic formation of the song control regions of the brain within the VZ [52]. Thus, the murine *Rps7* mutant phenotypes add to a growing body of literature linking individual RPs to specific features of neural development and suggesting that other RP mouse mutants may have previously uncharacterized defects in brain development.

Important questions remain regarding what underlying cellular mechanisms explain the similarities and differences among RP-associated phenotypes. A growing body of literature suggests a common mechanism where alterations in RP expression result in activation of TRP53, suggesting ribosomal stress is one of the many cellular anomalies detected by TRP53. For example, in their study of RP-mediated dark skin, McGowan and colleagues show that TRP53 acts as a sensor of ribosomal integrity, and that mutations in *Rps19* and *Rps20* can be phenotypically suppressed by *Trp53* deficiency [5]. *Trp53* haploinsufficiency also relieves the abnormal phenotypes of mice heterozygous for the sooty foot ataxia allele of *Rpl27a* [7] or the *Bst* allele of *Rpl24* [6]. Similarly, our results show that loss of *Rps7* increases TRP53 levels and that *Trp53* haploinsufficiency suppresses all morphological aspects of the *Rps7* phenotype. Our *in vivo* studies are supported by recent studies showing that *Rps7* depletion *in vitro* as well as *in vivo* in zebrafish induces TRP53 expression [53], [54], and collectively suggest the mechanism underlying the *Rps7* phenotype is TRP53 activation followed by TRP53-mediated apoptosis.

While these data suggest that RP mutant phenotypes may all be attributed to alterations in RP—TRP53 interactions, the variability among RP phenotypes suggests more complexity, potentially as a result of RP gene-specific or cell-specific functions [55]. Tissue-specific effects of mutations in different RPs have been observed in zebrafish [4], mouse [5], [6] and human [11]. These apparently tissue-specific phenotypes could simply be due to incomplete phenotypic characterization, differing severity of hypomorphic alleles, and/or differing genetic backgrounds. However, if they truly reflect tissue-specific RP functions, one explanation could be differences in the relative expression levels of individual RPs, with a consequent differential sensitivity to RP insufficiency that could vary with tissue type, developmental stage or differentiation state. Indeed, remarkable heterogeneity in RP mRNA levels has been observed in a variety of tissues [56] including specifically during mouse embryonic development [47] and human neuronal differentiation [57].

A cell type-specific sensitivity to RP insufficiency is strongly supported by the observation that keratinocyte-specific *Rps6* hemizygosity causes hyperpigmentation while melanocyte-specific *Rps6* hemizygosity instead causes hypopigmentation [5]. Similarly, the severe melanoblast reduction in *Rps7* mutants could suggest a melanocyte-specific sensitivity to RPS7 loss. Given that *Sox10* and *Rps7* pathways interact in melanocytes *in vivo* (Figure S6, Figure S7), it will be interesting to determine if a melanocyte-specific transcriptional response to RPS7 deficiency is mediated by *Sox10*, which is expressed in melanocytes but absent in keratinocytes. TRP53 activation in melanocytes has already been associated with downregulation of another white spotting gene, *Kit*, which is proposed as a transcriptional target of TRP53 [7], [58]. Further work is needed in melanocytes and other cell types that display RP gene-specific phenotypes, to clarify if direct transcriptional regulation of lineage-specific genes plays a role in tissue-specific sensitivity to individual RP deficiencies.

Another explanation of tissue-specific RP phenotypes could be a function for individual RPs in the translation of specific mRNAs. This is supported by studies of RPL13 [59] and by a recent study reporting patterning defects in *Rpl38* mutant mouse embryos due to RPL38 translational regulation of a subset of *Hox* transcripts [47]. The tissue-specific phenotypes observed in *Rps7* mutants would be consistent with a role for RPS7 in facilitating translational regulation of transcripts critical for development of the affected tissues. Alternatively, tissue-specific phenotypes observed in *Rps7* mutants could be a consequence of a direct interaction between RPS7 and tissue-specific developmental regulators at the protein level. Interestingly, support for protein-protein interactions between RPS7 and SOX10 comes from recent evidence that RPS7 interacts directly with SRY, an HMG box protein related to SOX10 [60]. This mechanism of direct interaction between RPs and tissue-specific, non-ribosomal proteins could explain tissue-specific phenotypes in RP mutants, and is supported by a number of studies reporting extra-ribosomal functions of specific RPs in regulation of ribosome biosynthesis and binding of transcription factor complexes (reviewed in ref. [55]).

The relative contributions of these various mechanistic explanations for tissue-specific and gene-specific RP mutant phenotypes will be clarified with further detailed comparison of mutants on consistent genetic backgrounds and with additional information from tissue-specific conditional alleles. In summary, these novel alleles of *Rps7* add to the growing collection of mammalian ribosomal mutants and provide two new mouse models of a DBA-associated gene. Importantly, the unique CNS apoptosis and behavioral phenotypes reported here suggest that RPs need to be considered as candidate genes for not only DBA but also a broad spectrum of neurodevelopmental human diseases.

## Materials and Methods

### Ethics statement

All procedures performed in the UK were in accordance with the UK Animals (Scientific Procedures) Act 1986, and those performed in the United States were approved by the IACUC in accordance with NIH guidelines.

### Mice

Montu mice were identified in a screen where BALB/c male mice were mutagenized using ENU and crossed to C3H/He for screening. Zuma mice were identified in an ENU screen sensitized by Sox10 haploinsufficiency (*Sox10^LacZ/+^*), where BALB/cJ mice were mutagenized using ENU and crossed to C57Bl/6J mice for further pedigree analysis. *Trp53* null mice (B6.129S2-Trp53^tm1Tyj/J^, stock #002101) were purchased from JAX Mice. For behavioral studies, mice were housed in facilities with a 12∶12 light∶dark cycle at a temperature of 22±1°C with a 60–70% humidity. Upon weaning, mice were separated into single-sex littermate groups and food was available *ad libitum*.

For body weight analysis, each gender/genotype group represented in Figure 1B was weighed over 10 weeks (average N = 9 for each Figure 1B data point, range N = 2–19). A Student's t-test was used to compare wild-type to mutant within gender-matched groups at each age. Significance was assessed using the Bonferonni method to correct for multiple testing, with p<0.001 deemed significant. In females, all time points after 1 week were significantly different and in males all time points after 2 weeks were significantly different, with the exception of 8.5 weeks where only 2 mutant mice were weighed (p<0.003).

Alizarin red and alcian blue staining of skeletons was conducted using standard techniques. Briefly, embryos were skinned and eviscerated, fixed 24 hours in ethanol, then fixed 24 hours in acetone. Staining was performed for 3–4 hours at 37°C and then 3–4 days at room temperature. After staining, embryos were rinsed in water and cleared in 1% KOH for 3 hours at room temperature and moved to fresh 1% KOH overnight. After clearing, embryos were serially transferred through 20%/1% KOH, 50% glycerol/1% KOH, and 80% glycerol/1% KOH.

FACS analysis was completed using standard techniques. Briefly, fetal liver samples were collected in ice cold PBS and dispersed into a single cell suspension with passage through a 21 gauge needle. Samples were incubated for 20 minutes with the addition of 10 µl of each antibody (BD Parmingen Ter119-APC and CD71-FITC), then washed once with PBS before FACS analysis. At each age ANOVA analysis was performed with a post-hoc test to compare selected pairs: (*Rps7*+/+ versus *Rps7^Zma^/+*) and (*Rps7^Zma^/+* versus *Rps7^Zma^/+*; *Trp53^KO^/+*).

### Genetic mapping and sequencing

DNA was extracted from mice using standard techniques. For *Mtu*, the mutation was mapped using 11 affected animals and 51 polymorphic MIT makers that were amplified by PCR and visualized on a 4% agarose gel stained with ethidium bromide. Fine mapping was performed with 13 additional SNPs that distinguished between BALB/c and 101. Seventeen known and predicted genes were identified in the *Mtu* critical interval from the Ensembl database (ENSMUSG00000020633, ENSMUSG00000066544, ENSMUSG00000020636, ENSMUSG00000036655, ENSMUSG00000061477, ENSMUSG00000020630, ENSMUSG00000020629, ENSMUSG00000020628, ENSMUSG00000036613, ENSMUSG00000061911, ENSMUSG00000020674, ENSMUSG00000020673, ENSMUSG00000020672, ENSMUSG00000043061, ENSMUSG00000044573, ENSMUSG00000020669, ENSMUSG00000036136). All exons and adjacent splice sites for these 17 genes were sequenced using a BigDye dideoxy-terminator system and analyzed on an ABI3700 sequencer (Applied Biosystems). For *Zma*, DNA from 8 offspring was analyzed using the Illumina GoldenGate assay medium density linkage panel (Illumina, San Diego, CA). The addition of 24 microsatellite markers, from 4 regions with suggestive linkage (Chr 4, 6, 12, 17) in 8 additional affected mice, allowed localization to chromosome 12 (D12Mit182 -D12Mit60). *Rps7* was sequenced as a candidate gene using the same methods as for *Mtu*.

### Cellular studies and Western blots

mRNA was extracted from wild-type and *Rps7^Mtu^/+* brains, and cDNA was prepared using reverse transcriptase (Invitrogen). A high fidelity PCR polymerase (KOD, Novagen) was then employed to amplify the *Rps7* transcript encoding a fusion protein tagged at either the C- or N-terminus with a FLAG epitope, prior to cloning into the pcDNA3.1(+) vector. Constructs were transfected into HeLa cells grown on glass coverslips, before fixation and staining 48 hours later. The primary antibody employed was a rabbit anti-FLAG (Abcam, ab21536, 1∶1000), followed by a fluorescent secondary (1∶200, Molecular Probes). Five independent transfections were performed. Images were captured on a Zeiss LSM 510 confocal microscope.

For Western blot experiments, DNA constructs encoding wild-type RPS7, RPS7 p.V156G, and RPS7 p.Y177S fused to the FLAG epitope at the N- or C-terminus were used in transient transfection experiments in human embryonic kidney (HEK) 293 cells. Neomycin phosphotransferase II (NPT2) expressed by the cloning vector pcDNA3.1 was used as a control for transfection efficiency. After transfection, proteins were separated on a 12% SDS-PAGE, transferred onto a nitrocellulose Protran membrane (Schleicher and Schuell) and incubated with a rabbit anti-FLAG or anti-neomycin phosphotransferase II (NPT2) antibody for 1 hr. Following three consecutive washes in PBS/Tween, the membrane was incubated with horseradish peroxidase-conjugated goat anti-rabbit Ab (Jackson Immunoresearch) and visualized using a chemiluminescence detection kit (Pierce) and a LAS3000 Image System (Fuji). Equal loading was checked by Ponceau-S red staining of the membranes before Western blot analysis.

### Ribosomal analysis

For Northern blots for rRNA processing, RNA was extracted from mouse tissues with Trizol (Invitrogen) according to the manufacturer's protocol. Total RNA was fractionated on formaldehyde-agarose gels and transferred to Gene Screen Plus membrane (NEN). To detect rRNA precursor transcripts, ITS1 probe was prepared by 5′ end-labeling of a 28-mer oligonucleotide (GCTCCTCCACAGTCTCCCGTTAATGATC) with 25 µCi of γ [32P]-ATP and T4 polynucleotide kinase according to standard protocols. Hybridization was carried out overnight at 42°C in 6× SSPE, 1% SDS, 0.25 mg/ml ssDNA and 5× Denhardt's solution. After hybridization, the blot was washed twice with 1% SDS in 2× SSPE for 30 min at 37°C. Quantitation of signals was obtained by phosphor screen scanning with a STORM PhosphorImager and ImageQuant software analysis (Molecular Dynamics). The experiments were performed on tissues from *Rps7*+/+ and *Rps7^Mtu^/+* littermate mice (2 *Rps7*+/+ and 2 *Rps7^Mtu^/+* for liver, 3 *Rps7*+/+ and 3 *Rps7^Mtu^/+* for brain, each in triplicate). Quantitation is reported as the ratio between 30S and 21S rRNA precursors and was significantly different between genotypes in both brain (p<0.01) and liver (p<0.01) (two-way ANOVA with post hoc t-test).

For ribosome isolation, cytoplasmic extracts from RPS7 p.V156G transiently transfected HEK-293 cells were fractionated through ultracentrifugation on a sucrose cushion. After 2 hours of centrifugation at 100,000 g, ribosomes and ribosomal subunits were recovered in the pellet and free cytoplasmic proteins were recovered from the supernatant.

For sucrose gradients separating polysomes and ribosomal subunits, cytoplasmic extracts from *Mtu/+* liver were loaded onto a 10%–30% linear sucrose gradient containing 30 mM Tris-HCl (pH 7.5), 100 mM NaCl, and 10 mM MgCl_2_. Gradients were centrifuged in a Beckman SW 41 rotor for 5 hr at 37,000 rpm and then the absorbance profile of the gradient was used to evaluate the ratio between the peaks of ribosomal subunits (60S and 40S).

### *In situ* hybridization

For whole mount, *Pmel17*-containing plasmid (Riken cDNA clone G370069C13; GenBank Acc: BB766987) was digested with Kpn1 and transcribed with T3 polymerase to generate DIG-labeled *in situ* probes. Hybridizations were performed using published protocols [61] with the following modifications. After probe hybridization, Ribonuclease A digestion was omitted, and Tris-buffered saline was used in place of PBS. BM-purple substrate (Roche, Molecular Biochemicals) was used in place of 5-bromo-4-chloro-3-indolyl phosphate/nitroblue tetrazolium. Embryos were photographed using a Zeiss SteREO Discovery V12 microscope with a Zeiss AxioCam camera. Probes to detect *Rps7* by *in situ* hybridization were amplified by PCR and cloned into the pCRII- TOPO vector (Invitrogen) using the following primer pairs: R7_IN_F AAGGAAATCGAAGTTGGTG and R7_IN_ R AATTAACATCCTTGCCTGTG. Mouse embryos were fixed in 2–4% (w/v) paraformaldehyde, cryoprotected with 30% (w/v) sucrose in phosphate buffer before sectioning (10–20 µm) on a cryostat. *In situ* hybridization was carried out as previously described [62]. Briefly, hybridization of sections with ^35^S-UTP-labeled RNA probes was carried out in a 50% (v/v) formamide solution at 60°C. Sections were washed in 50% (v/v) formamide, an RNAse A treatment was performed for 30 min at 37°C, and then successively stringent SSC solution washes were performed, with a final wash at 0.1× SSC at 60°C.

### Histology and immunohistochemistry

For Nissl staining, adult mice were perfused with 0.9% NaCl and 4% (w/v) paraformaldehyde before the brain was dehydrated in 30% sucrose, sectioned (40 µm) on a freezing microtome, and stained.. For embryonic histology, embryos were fixed in either Bouin's fixative or 4% paraformaldehyde overnight, washed extensively in PBS, and dehydrated in 70% ethanol before sectioning (5–7 µm paraffin) and hematoxylin and eosin (H and E) staining. For immunohistochemistry, embryos were fixed in 4% paraformaldehyde overnight, washed with PBS, dehydrated in 10% sucrose followed by 20% sucrose, then embedded in Neg-50 (Thermo Scientific) for cryosectioning (14 µm). Antibodies included anti-TRP53 (Leica NCL-p53-CM5p, 1∶200), anti-CC3 (Cell Signaling #9661, 1∶200), and anti-PH3 (Millipore #06-570, 1∶200) and resulting stains were imaged using a Zeiss Observer.D1 microscope. For apoptosis and proliferation experiments, transverse cryo-sections collected at the level of the forelimb from 3 embryos of each genotype, 6 sections per embryo, were counted for CC3+ and PH3+ cells. InStat software (GraphPad Software, Inc.) was used for one-way ANOVA statistical analysis. No significant difference among the three genotypes was observed in PH3+ cell counts (p = 0.902). CC3 counts were significantly different among genotypes (p = 0.0005) and a Tukey-Kramer multiple comparison post test was used for pairwise comparison among all genotypes with the following results: *Rps7*+/+ versus *Rps7^Zma^/+* (p<0.001), *Rps7*+/+ versus *Rps7^Zma^/+*; *Trp53^KO^/+* (P>0.05, no significant difference), *Rps7^Zma^/+* versus *Rps7^Zma^/+*; *Trp53^KO^/+* (p<0.01).

### Behavioral phenotyping

Behavioral phenotyping was performed on mutant and unaffected sex-matched littermates between 8 and 12 weeks of age. Open-field tests were conducted for 5 minutes in a brightly lit, 60 cm diameter, enclosed white arena (field), and monitored by an automated tracking system to measure the total distance travelled and the amount of time spent in the center versus the border of the field. The elevated plus maze test was conducted with an elevated platform consisting of two enclosed arms and two open arms. An automated tracking system was employed to measure the number of entries into each arm and distance traveled in open and closed arms of the elevated plus maze over 5 minutes. Spontaneous alternation was performed in an enclosed T-maze built from gray plastic (arm dimensions 30 cm×10 cm×30 cm). Each mouse underwent 10 trials with an inter-trial interval of at least 20 minutes. For each trial the mouse was placed in the start arm facing the end wall and allowed to enter a goal arm of its own choice. The mouse was confined in the goal arm for 30 seconds and then put back in the start arm and allowed a free choice of either arm [31].

### MRM imaging

Late gestation (E18.5) embryos were collected, fixed in 4% paraformaldehyde overnight, washed extensively in PBS, and stored in 70% ethanol. Prior to imaging, embryos were rehydrated in a solution of PBS and 0.5% (v/v) Magnevist (Bayer Healthcare Pharmaceuticals, Wayne, NJ), a contrast agent. MRM was performed on specimens using a Bruker 14.1T MR imaging spectrometer (Bruker Biospin, Billerica, MA) using a multi-echo RARE technique [63] with TR/TE = 200/6.9 ms, 8-echoes, and 4 signal averages. The resulting 3D images were acquired in 53 min with an acquisition resolution of 50 microns isotropic. Manual segmentation of the whole brain and 10 major anatomical regions was performed using Amira visualization software (v5.2.2, Visage Imaging Inc., Andover MA, USA) with guidance from an anatomical reference atlas [64]. The volume of these regions was estimated and 3D rendering with smoothing was performed to generate 3D representations. Student's t-tests were used to compare volumes of each *Rps7*+/+ and *Rps7^Zma^*/+ brain region. Significance was assessed using the Bonferonni method to correct for multiple testing, with p<0.005 deemed significant.

## Supporting Information

Figure S1 Montu and zuma map to mouse Chromosome 12 and cause a variety of phenotypes. (A,B) Genotyping data for montu (*Mtu*, A) and Zuma (*Zma*, B) mapping. Black boxes represent heterozygous genotypes and white boxes represent homozygous wild-type genotypes. Marker names are listed at left and the number of mice in each genotype category is shown beneath each column. Double-headed arrows at right indicate the critical interval that flanked a non-recombinant marker. (C) The *Mtu* and *Zma* critical intervals, markers used for mapping, and relevant map intervals on mouse Chromosome 12 are indicated. Coordinates are based on NCBI Build 34. (D) Results from an *Rps7^Mtu^*×*Rps7^Zma^* intercross showed that *Rps7^Mtu^* and *Rps7^Zma^* do not complement each other and are therefore alleles of the same gene.
(TIF)

Figure S2 Evolutionary conservation of RPS7 and predicted structural effects of *Rps7^Mtu^* (p.V156G) and *Rps7^Zma^* (p.Y177S) mutations. (A) The amino acid residues altered by the *Rps7^Mtu^* and *Rps7^Zma^* mutations are highly conserved across a wide range of species. Green highlight indicates the location of p.V156 and p.Y177, and red font indicates non-identical residues. (B) Alignments of secondary structure predictions indicate that the *Rps7^Mtu^* and *Rps7^Zma^* mouse alleles cause no gross structural changes in RPS7 as a result of the encoded amino acid alterations. The experimentally determined secondary structures of yeast (*S. cerevisiae*) and tetrahymena (*T. thermophila*) RPS7 orthologs (PDB ID 3U5C and PDB ID 2XZM, respectively) are aligned with secondary structure predictions of the *S. cerevisiae*, *T. thermophila*, and mouse RPS7, RPS7^Mtu^ and Rps7^Zma^ proteins (generated with both PSSpred and PROFsec). In the predictions, numbers indicate reliability scores ranging from low (0) to high (9). Residues predicted as helix and strand are highlighted in blue and gray, respectively. (C) The 3-dimensional structure of the yeast 80S ribosomal subunit [21]. Color-coding is as follows: RPS7, red; 60S subunit proteins, cyan; 40S subunit proteins, purple; and rRNA, gray. The position of residues homologous to mouse p.V156 and p.Y177 are highlighted in green. (D) Closer view of the RPS7-containing region of the 80S side view shown in panel C.
(TIF)

Figure S3 Mutant RPS7 is assembled into ribosomes. (A) Western blot showing similar levels of expression for N- and C-terminal FLAG tagged wild-type RPS7 (WT N and WT C, respectively) and RPS7^Zma^ (MUT N and C, respectively) in HEK-293 cells. NPT2 expression is shown as a control to normalize for transfection efficiency. (B) Cytoplasmic extracts from transiently transfected HEK-293 cells were fractionated. A similar fraction of all transfected RPS7 proteins is observed in the ribosomal pellet (P) compared to the supernatant (S). (C) Polysomal profiles of liver cytoplasmic extracts. After ultracentrifugation, the Optical Density at 260 nm was measured along the sucrose gradient. Ratio between 60S and 40S peaks in *Rps7^Mtu^/+* is similar to control. The slight variation in 80S peak relative height is not significant as it is within the variability observed between samples, independent of genotype.
(TIF)

Figure S4 Embryonic developmental delay in *Rps7^Zma^*/+ mice is suppressed by *Trp53* deficiency. Chronologically age-matched littermate embryos illustrate that *Rps7^Zma^/+* mice exhibit developmental delay at E11.5 and E12.5 relative to *Rps7*+/+ littermates (left 4 panels). This *Rps7^Zma^-*associated developmental delay is suppressed by *Trp53* deficiency in *Rps7^Zma^/+; Trp53^KO^/+* (right 4 panels). Images for each of the 4 genotypes are shown at the same magnification within an age (row).
(TIF)

Figure S5 *Pmel17* expression is reduced at E12.5 in *Rps7* mutant embryos. Comparison of *Pmel17 in situ* hybridizations of E12.5 *Rps7^Zma^/+* embryos (middle 3 panels) with E12.5 *Rps7*+/+ embryos (left 3 panels) showed that *Rps7^Zma^/+* embryos exhibit a severe reduction in melanoblasts. Furthermore, melanoblast number in E12.5 *Rps7^Zma^/+* embryos was reduced below that of E11.5 *Rps7*+/+ embryos (right 3 panels), thus showing that the melanoblast reduction in *Rps7^Zma^/+* embryos exceeds the gross developmental delay observed in *Rps7^Zma^/+* mutants. This reduction is especially evident from comparison of E12.5 *Rps7^Zma^/+* and E11.5 *Rps7*+/+ melanoblast populations in the otic area (arrows), in the trunk posterior to the forelimb (*), and in the tail region (arrowheads). Scale bars = 1 mm.
(TIF)

Figure S6 Mutation of *Rps7* and *Sox10* act synergistically to reduce melanoblast number. Combined haploinsufficiency for *Rps7* and *Sox10* (*Rps7^Zma/^+; Sox10^LacZ/^+* double heterozygotes) greatly reduced melanoblast number at E12.5 (middle 3 panels) as compared to E12.5 *Rps7*+/+; *Sox10^LacZ/^+*mice (left 3 panels). Furthermore, E12.5 *Rps7^Zma/^+; Sox10^LacZ/^+* mice showed a greater reduction in melanoblast number in comparison with E11.5 *Rps7*+/+; *Sox10^LacZ/^+* mice, indicating that this reduction in melanoblasts exceeded the gross developmental delay observed in *Rps7^Zma^/+* mutants. Reduction is especially evident in melanoblast populations in the otic area (arrow), and in the tail region posterior to the hindlimb (arrowhead and posterior). Scale bars = 1 mm.
(TIF)

Figure S7 *Rps7* mutation increases *Sox10*-dependent hypopigmentation and does not result in dark skin. (A, B) *Rps7^Zma/^+; Sox10^LacZ/^+* double mutants that survive postnatally exhibit more extensive hypopigmentation than *Rps7^Zma/^+*or *Sox10^LacZ/^+* mutants. A representative *Rps7^Zma/^+; Sox10^LacZ/^+* mouse is shown (A) along with quantitative scoring (0 = no spotting; 4 = the largest ventral spots that extend to the dorsal surface) comparing the white spotting in *Rps7*+/+; *Sox10^LacZ/^+* (blue, N = 27), *Rps7^Zma/^+; Sox10*+/+ (red, N = 16), and *Rps7^Zma/^+; Sox10^LacZ/^+* (yellow, N = 8) (B). (C, D) Adult *Rps7* mutants do not have dark skin in foot pads and tails. (E,F) H&E stained sections through the tail skin of *Rps7^Zma/^+* mice reveal epidermal (*) and dermal (arrowhead) pigmentation similar to that of *Rps7*+/+ mice, confirming the presence of melanocytes in the tail.
(TIF)

Figure S8 Behavioral assessment of *Rps7^Mtu^/+* mutants. (A) Total distance traveled in the open-field test by *Rps7*+/+ controls (WT) and *Rps7^Mtu^/+* (Mtu) (N = 11) was similar (p = 0.5). (B–E) Likewise there were no significant differences between *Rps7*+/+ and *Rps7^Mtu^/+* on the elevated plus maze. Open arm distance (B, P>0.5), open arm entries (C, P>0.5), closed arm distance (D, P>0.5), and closed arm entries (E, P>0.1) were all comparable (N = 11).
(TIF)

Figure S9 *Rps7* mutants have pyknotic nuclei in the spinal cord. H&E stained sagittal sections through the spinal cord of E18.5 *Rps7*+/+ (A, B) and *Rps7^Zma/+^* (C, D) embryos. Arrow in D indicates an example of a darkly stained, condensed nucleus characteristic of pyknotic nuclei observed throughout the *Rps7^Zma/+^* spinal cord. Anterior is oriented towards the top and posterior to the bottom of all images. Boxed regions in A, C define the respective areas shown at higher magnification in B, D. Abbreviations: vertebral column (V), spinal cord (SC). Scale bars = 100 µm.
(TIF)

Figure S10 *Rps7* mRNA expression pattern. Mouse *Rps7* transcript was detected by *in situ* hybridization at E13.5 (A, E), E16.5 (B, F), E17.5 (D), and adulthood (C, G). Broad expression was detected in the developing embryo, including a high level of expression in the ganglionic eminences (arrowhead in D), the proliferative ventricular zone (E), as well as in the dentate gyrus on the hippocampus in adult animals (G). Abbreviations: ventricular zone/subventricular zone (VZ/SVZ), intermediate zone (IZ), cortical plate (CP), CA3 region of hippocampus (CA3), dentate gyrus (DG).
(TIF)

Figure S11 *Rps7* mutants have increased TRP53 expression. (A–C) Increased TRP53 expression was detected in E11.5 *Rps7^Zma^/+* (Z/+) coronal sections through the neocortex compared to *Rps7*+/+ (+/+) and *Rps7^Zma^/+*; *Trp53^KO^/+* (Z/+;p53/+). (D–I) This increase was also detected in the neural tube of *Rps7^Zma^/+* embryos at E11.5 (D–F) and E12.5 (G–I). Scale bars = 100 µm.
(TIF)

Table S1 Circulating blood counts from+/+and *Rps7^Mtu^/+* adult male mice.
(PDF)

Table S2 Biochemical panel from+/+and *Rps7^Mtu^/+* adult male mice.
(PDF)

Table S3 *Rps7^Zma^/+* erythrocytes are able to terminally differentiate, but they display modest developmental delay that is suppressed by *Trp53* haploinsufficiency.
(PDF)

Table S4 Surviving numbers of postnatal offspring from an *Rps7^Zma/+^*; *Trp53^+/−^*×C57BL/6J cross demonstrate that *Trp53* haploinsufficiency restores *Rps7* viability.
(PDF)

Video S1 MRM-based reconstructions of *Rps7*+/+ brain. Regions of late gestation (E18.5) brain defined by manual segmentation of MRM images for volumetric calculations are shown as follows: olfactory bulbs (light gray), lateral ventricles (lime green), cortex (red), septum (yellow), striatum (dark blue), 4^th^ ventricle (charcoal gray), hippocampus (purple), thalamus (cyan), colliculi (orange), cerebellum (dark green).
(MOV)

Video S2 MRM-based reconstructions of *Rps7^Zma/^+* brain. Regions of late gestation (E18.5) brain defined by manual segmentation of MRM images for volumetric calculations are shown as follows: olfactory bulbs (light gray), lateral ventricles (lime green), cortex (red), septum (yellow), striatum (dark blue), 4^th^ ventricle (charcoal gray), hippocampus (purple), thalamus (cyan), colliculi (orange), cerebellum (dark green).
(MOV)
