# Correction: Mutation of the Diamond-Blackfan Anemia Gene *Rps7* in Mouse Results in Morphological and Neuroanatomical Phenotypes

**DOI:** 10.1371/journal.pgen.1005682

**Published:** 2015-11-19

**Authors:** Dawn E. Watkins-Chow, Joanna Cooke, Ruth Pidsley, Andrew Edwards, Rebecca Slotkin, Karen E. Leeds, Raymond Mullen, Laura L. Baxter, Thomas G. Campbell, Marion C. Salzer, Laura Biondini, Gretchen Gibney, Françoise Phan Dinh Tuy, Jamel Chelly, H. Douglas Morris, Johannes Riegler, Mark F. Lythgoe, Ruth M. Arkell, Fabrizio Loreni, Jonathan Flint, William J. Pavan, David A. Keays

The top left image of Fig 2B is incorrect. The authors have provided a corrected version of Fig 2 here.

**Fig 2:**
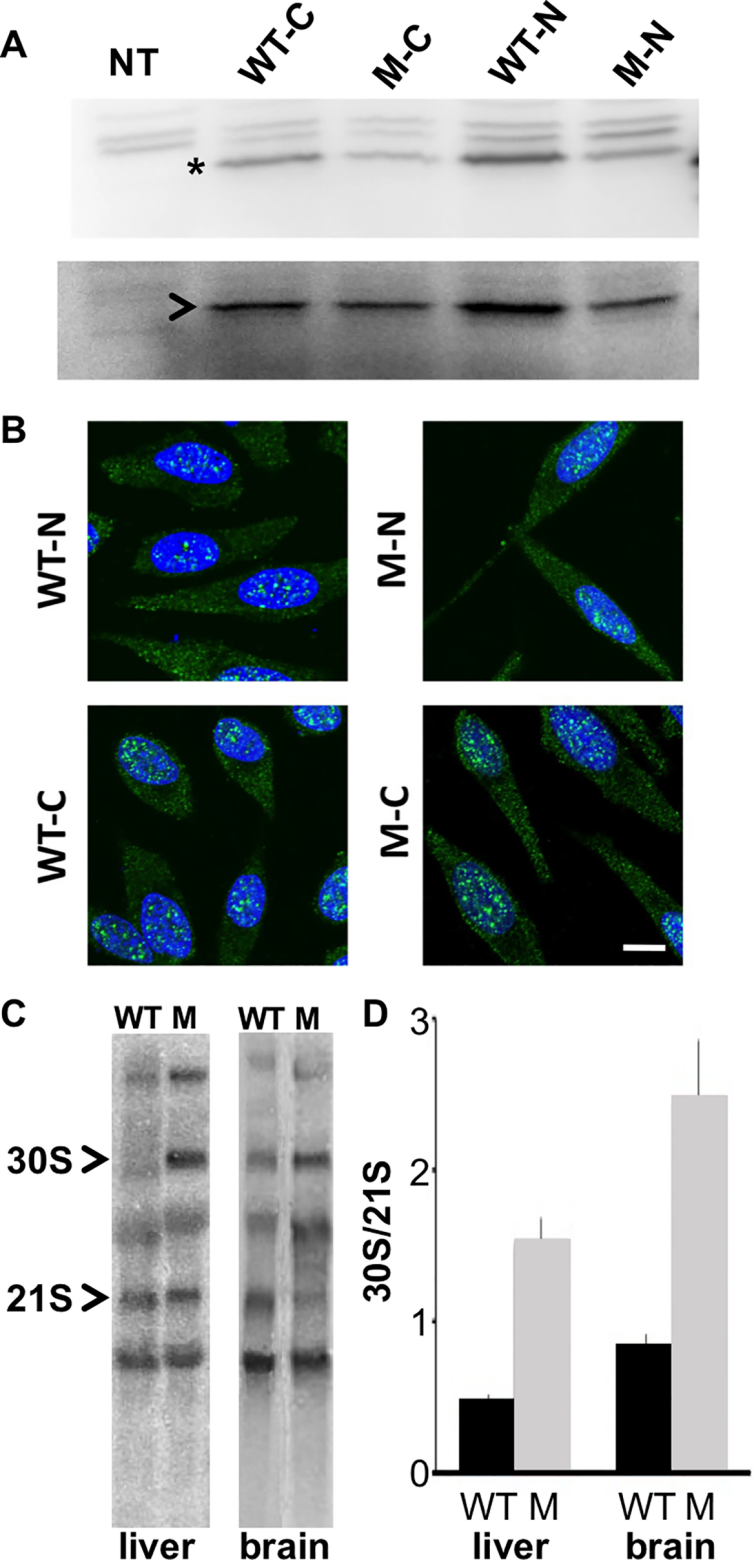
*Rps7* ^*Mtu*^ shows reduced function in ribosomal precursor processing. (A) Western blot showing similar levels of expression for N- and C-terminal FLAG-tagged wild-type RPS7 (WT-N and WT-C, respectively) and RPS7^Mtu^ (M-N and M-C, respectively) proteins in HEK-293 cells. The RPS7-specific band is indicated by *, and NPT2 (arrowhead) expression is shown as a control. (B) Subcellular localization of N- and C-terminal FLAG-tagged RPS7 proteins. Wild-type RPS7 and RPS7^Mtu^ both localize to speckles in the nucleus and are observed throughout the cytoplasm. Scale bar = 10 μm. All panels are at the same magnification. (C) Representative Northern blot analysis of liver and brain RNA from wild-type (WT) and *Rps7* ^*Mtu*^/+ (M) mice detecting various rRNA precursors using a probe within the internal transcribed spacer (ITS1). The 30S and 21S rRNA precursors are indicated. (D) Quantitation of the signals of Northern experiments reported as the ratio between 30S and 21S rRNA precursors was significantly different between *Rps7*+/+ and Rps7Mtu/+ (* indicates p<0.01). The average of the values is reported in the bar graphs with S.E.M.
