# Supplementary figures and images for: Mutation of the Diamond-Blackfan Anemia Gene Rps7 in Mouse Results in Morphological and Neuroanatomical Phenotypes
Source: PLoS Genet. 2013 Jan 31;9(1):e1003094. doi: 10.1371/journal.pgen.1003094 (PMC3561062; doi:10.1371/journal.pgen.1003094)

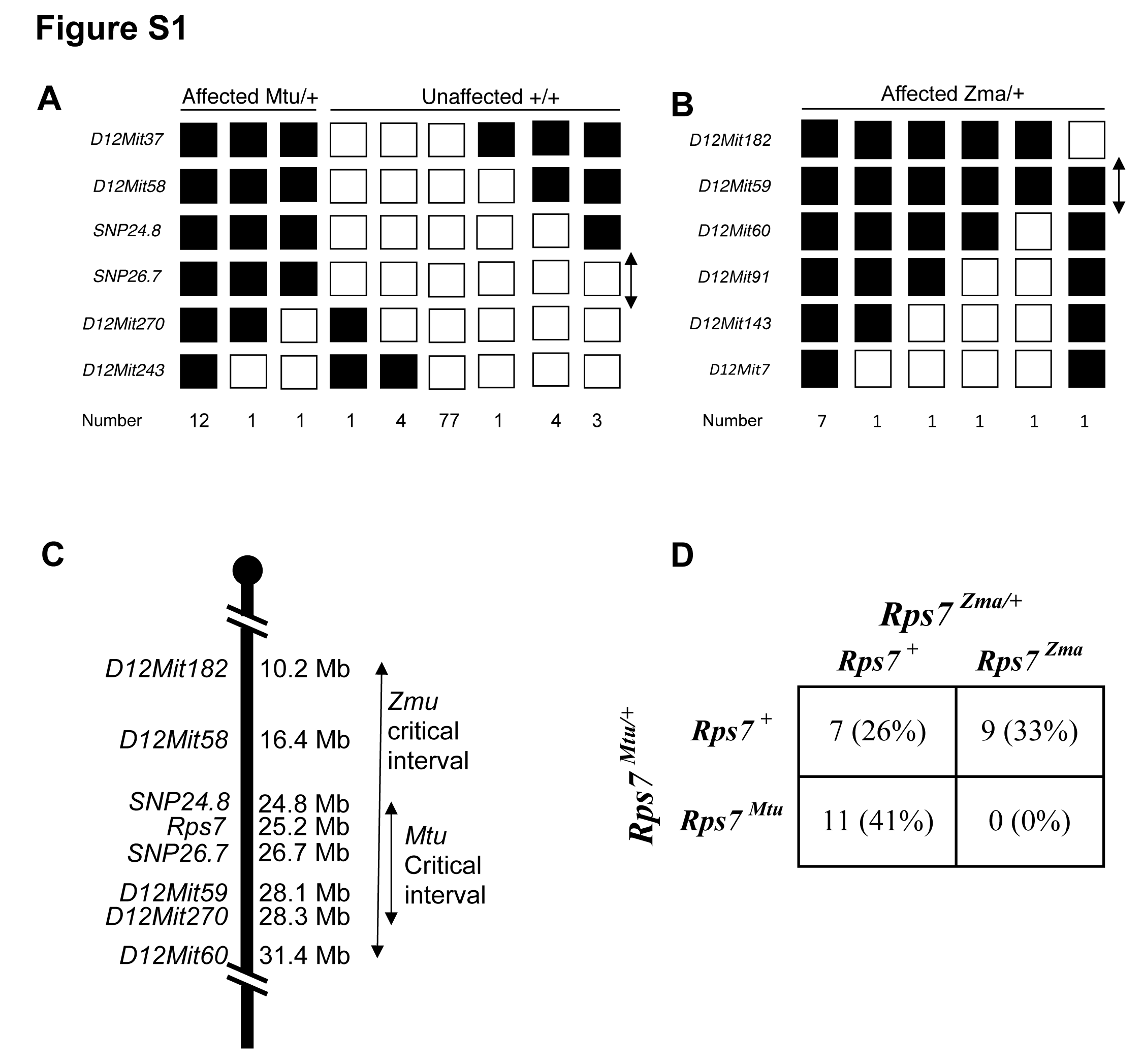

Supplement: Figure S1 — Montu and zuma map to mouse Chromosome 12 and cause a variety of phenotypes. (A,B) Genotyping data for montu (Mtu, A) and Zuma (Zma, B) mapping. Black boxes represent heterozygous genotypes and white boxes represent homozygous wild-type genotypes. Marker names are listed at left and the number of mice in each genotype category is shown beneath each column. Double-headed arrows at right indicate the critical interval that flanked a non-recombinant marker. (C) The Mtu and Zma critical intervals, markers used for mapping, and relevant map intervals on mouse Chromosome 12 are indicated. Coordinates are based on NCBI Build 34. (D) Results from an Rps7Mtu×Rps7Zma intercross showed that Rps7Mtu and Rps7Zma do not complement each other and are therefore alleles of the same gene. (TIF) [file pgen.1003094.s001.tif]

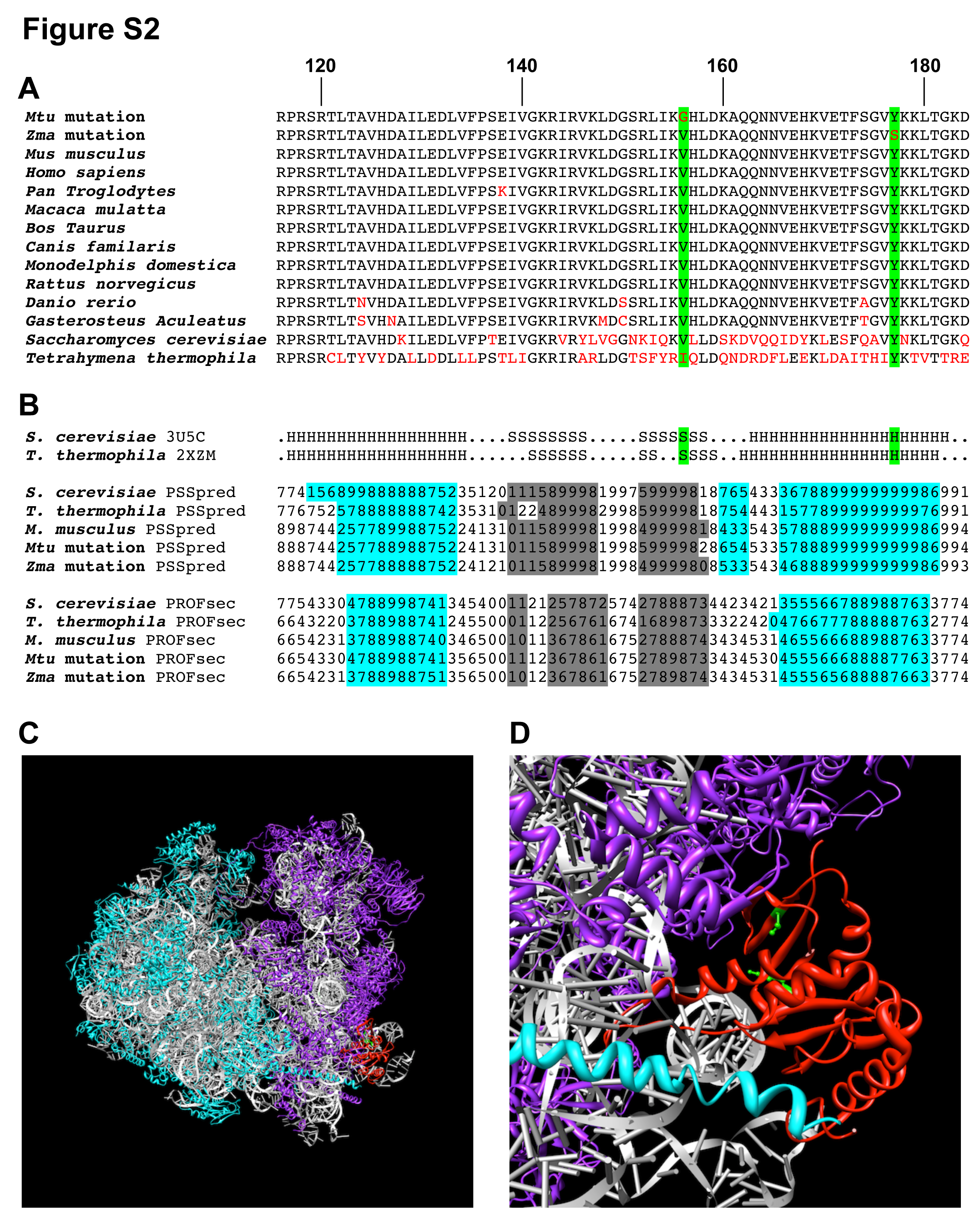

Supplement: Figure S2 — Evolutionary conservation of RPS7 and predicted structural effects of Rps7Mtu (p.V156G) and Rps7Zma (p.Y177S) mutations. (A) The amino acid residues altered by the Rps7Mtu and Rps7Zma mutations are highly conserved across a wide range of species. Green highlight indicates the location of p.V156 and p.Y177, and red font indicates non-identical residues. (B) Alignments of secondary structure predictions indicate that the Rps7Mtu and Rps7Zma mouse alleles cause no gross structural changes in RPS7 as a result of the encoded amino acid alterations. The experimentally determined secondary structures of yeast (S. cerevisiae) and tetrahymena (T. thermophila) RPS7 orthologs (PDB ID 3U5C and PDB ID 2XZM, respectively) are aligned with secondary structure predictions of the S. cerevisiae, T. thermophila, and mouse RPS7, RPS7Mtu and Rps7Zma proteins (generated with both PSSpred and PROFsec). In the predictions, numbers indicate reliability scores ranging from low (0) to high (9). Residues predicted as helix and strand are highlighted in blue and gray, respectively. (C) The 3-dimensional structure of the yeast 80S ribosomal subunit [21]. Color-coding is as follows: RPS7, red; 60S subunit proteins, cyan; 40S subunit proteins, purple; and rRNA, gray. The position of residues homologous to mouse p.V156 and p.Y177 are highlighted in green. (D) Closer view of the RPS7-containing region of the 80S side view shown in panel C. (TIF) [file pgen.1003094.s002.tif]

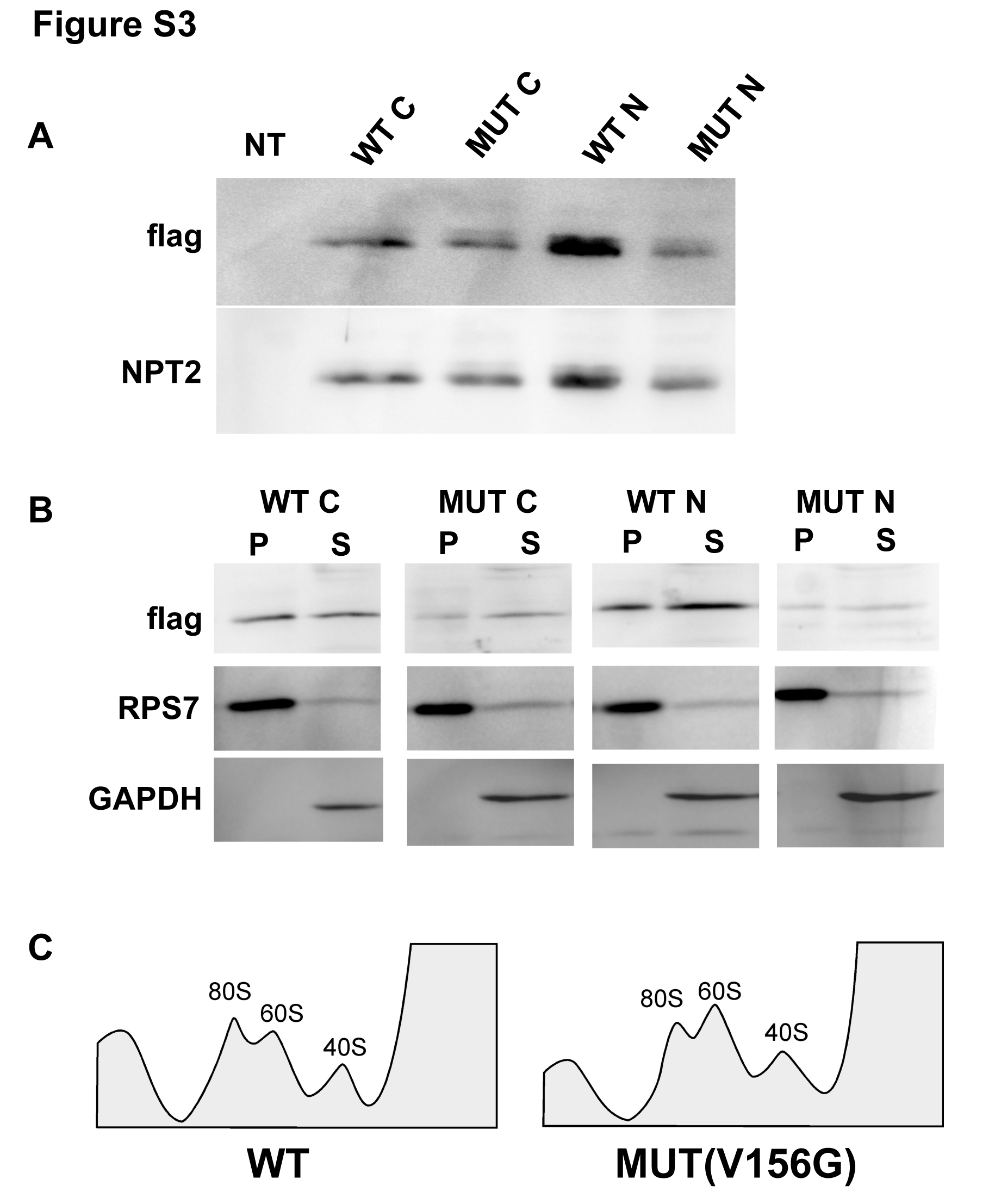

Supplement: Figure S3 — Mutant RPS7 is assembled into ribosomes. (A) Western blot showing similar levels of expression for N- and C-terminal FLAG tagged wild-type RPS7 (WT N and WT C, respectively) and RPS7Zma (MUT N and C, respectively) in HEK-293 cells. NPT2 expression is shown as a control to normalize for transfection efficiency. (B) Cytoplasmic extracts from transiently transfected HEK-293 cells were fractionated. A similar fraction of all transfected RPS7 proteins is observed in the ribosomal pellet (P) compared to the supernatant (S). (C) Polysomal profiles of liver cytoplasmic extracts. After ultracentrifugation, the Optical Density at 260 nm was measured along the sucrose gradient. Ratio between 60S and 40S peaks in Rps7Mtu/+ is similar to control. The slight variation in 80S peak relative height is not significant as it is within the variability observed between samples, independent of genotype. (TIF) [file pgen.1003094.s003.tif]

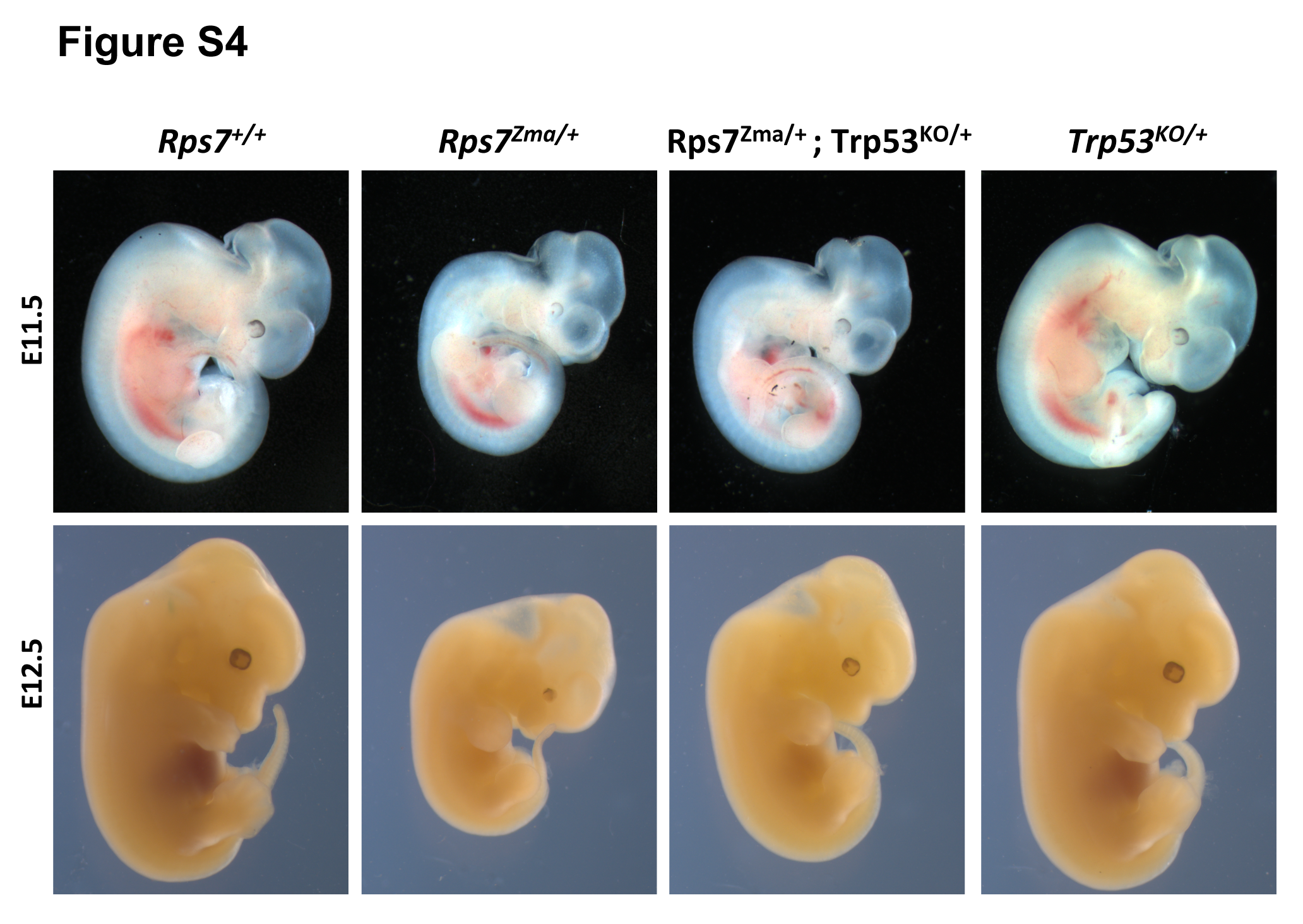

Supplement: Figure S4 — Embryonic developmental delay in Rps7Zma/+ mice is suppressed by Trp53 deficiency. Chronologically age-matched littermate embryos illustrate that Rps7Zma/+ mice exhibit developmental delay at E11.5 and E12.5 relative to Rps7+/+ littermates (left 4 panels). This Rps7Zma-associated developmental delay is suppressed by Trp53 deficiency in Rps7Zma/+; Trp53KO/+ (right 4 panels). Images for each of the 4 genotypes are shown at the same magnification within an age (row). (TIF) [file pgen.1003094.s004.tif]

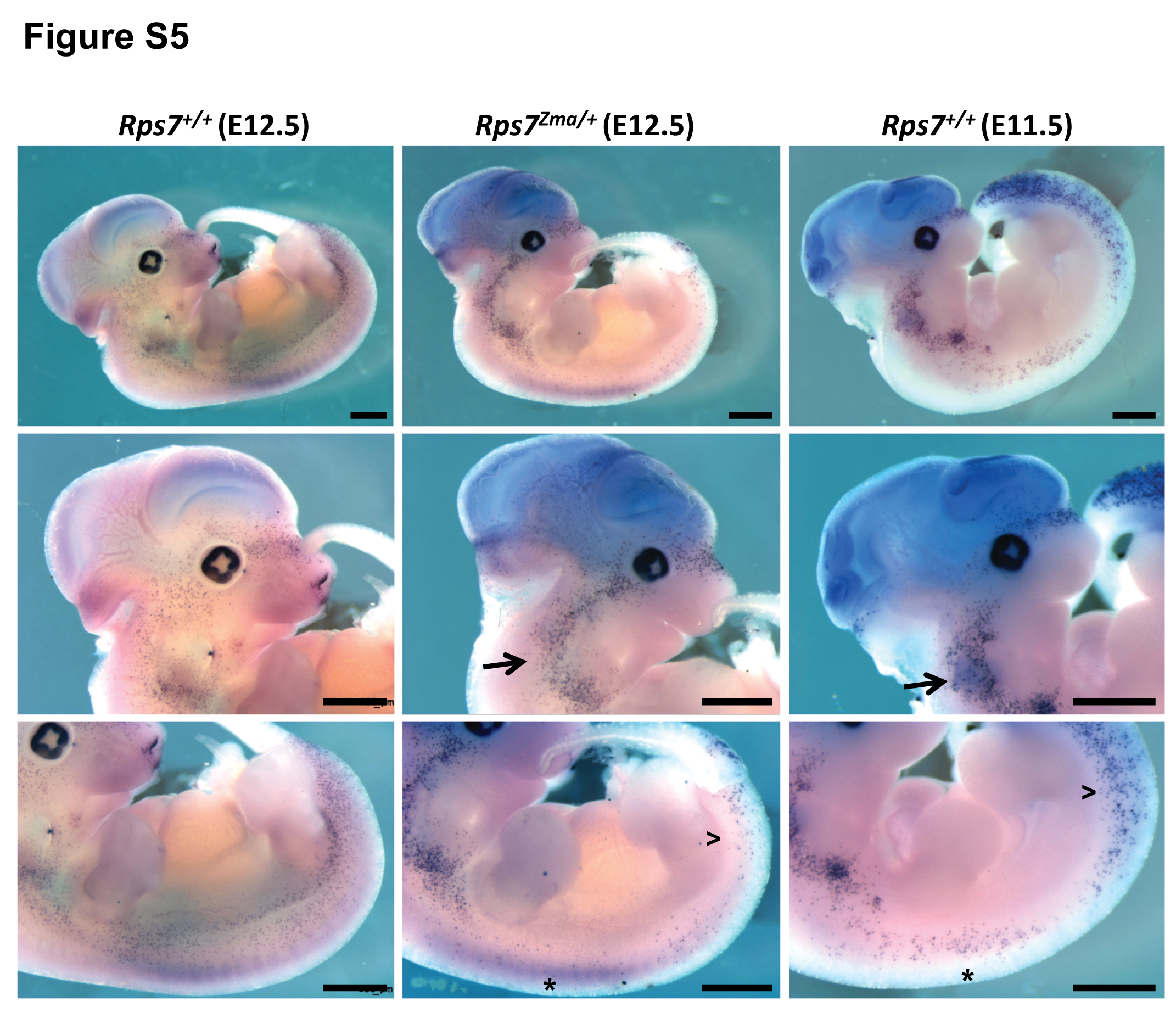

Supplement: Figure S5 — Pmel17 expression is reduced at E12.5 in Rps7 mutant embryos. Comparison of Pmel17 in situ hybridizations of E12.5 Rps7Zma/+ embryos (middle 3 panels) with E12.5 Rps7+/+ embryos (left 3 panels) showed that Rps7Zma/+ embryos exhibit a severe reduction in melanoblasts. Furthermore, melanoblast number in E12.5 Rps7Zma/+ embryos was reduced below that of E11.5 Rps7+/+ embryos (right 3 panels), thus showing that the melanoblast reduction in Rps7Zma/+ embryos exceeds the gross developmental delay observed in Rps7Zma/+ mutants. This reduction is especially evident from comparison of E12.5 Rps7Zma/+ and E11.5 Rps7+/+ melanoblast populations in the otic area (arrows), in the trunk posterior to the forelimb (*), and in the tail region (arrowheads). Scale bars = 1 mm. (TIF) [file pgen.1003094.s005.tif]

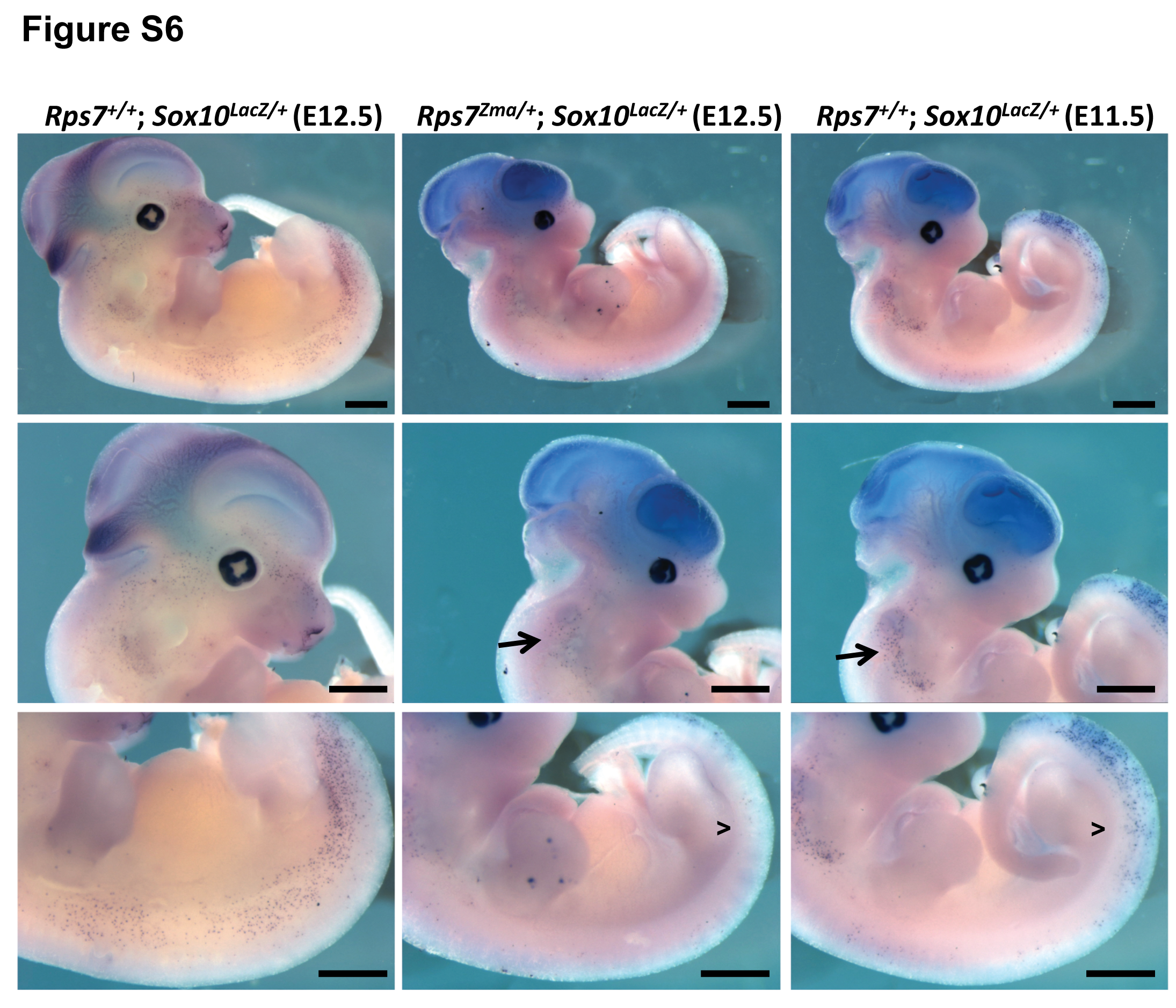

Supplement: Figure S6 — Mutation of Rps7 and Sox10 act synergistically to reduce melanoblast number. Combined haploinsufficiency for Rps7 and Sox10 (Rps7Zma/+; Sox10LacZ/+ double heterozygotes) greatly reduced melanoblast number at E12.5 (middle 3 panels) as compared to E12.5 Rps7+/+; Sox10LacZ/+mice (left 3 panels). Furthermore, E12.5 Rps7Zma/+; Sox10LacZ/+ mice showed a greater reduction in melanoblast number in comparison with E11.5 Rps7+/+; Sox10LacZ/+ mice, indicating that this reduction in melanoblasts exceeded the gross developmental delay observed in Rps7Zma/+ mutants. Reduction is especially evident in melanoblast populations in the otic area (arrow), and in the tail region posterior to the hindlimb (arrowhead and posterior). Scale bars = 1 mm. (TIF) [file pgen.1003094.s006.tif]

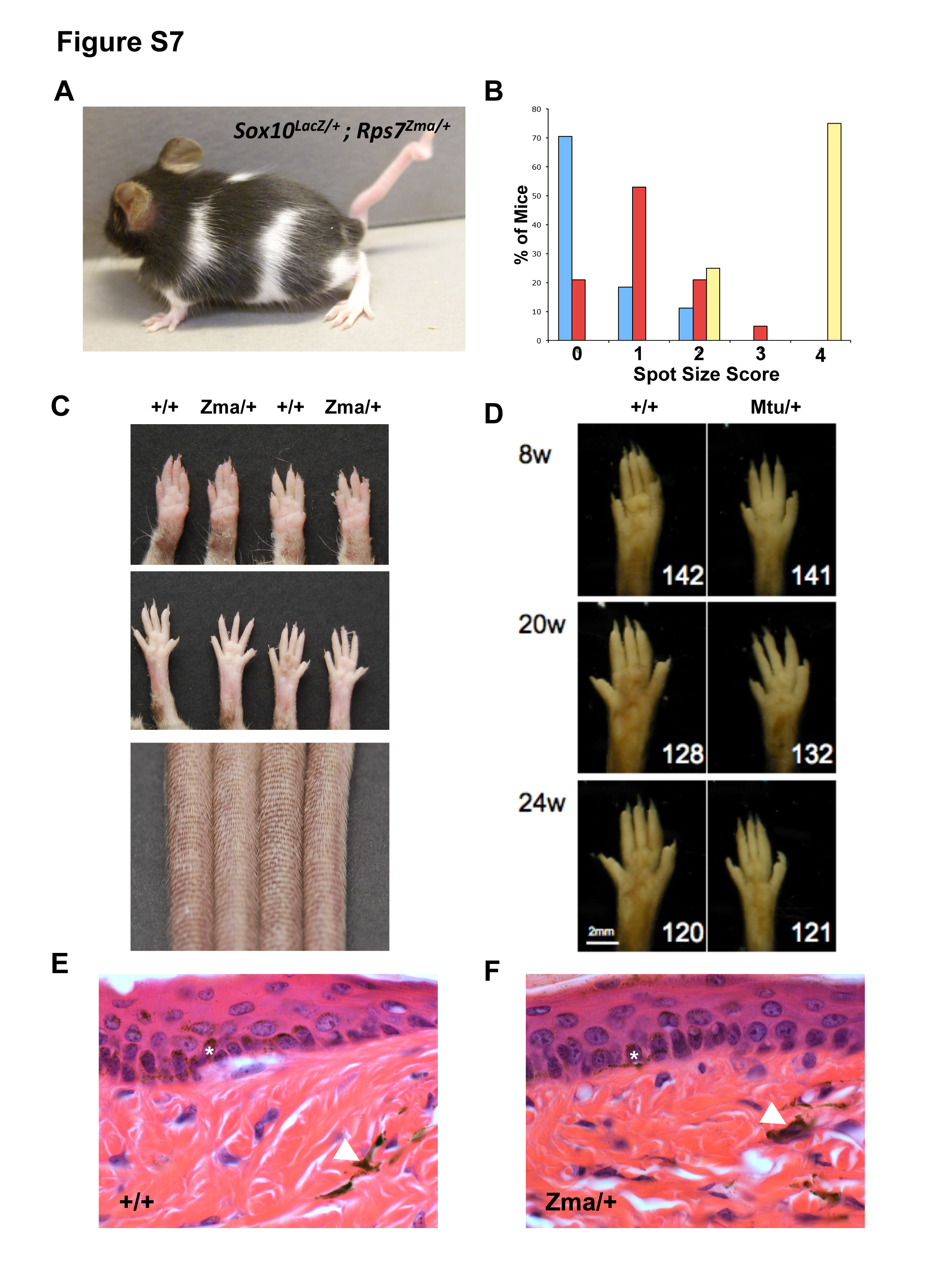

Supplement: Figure S7 — Rps7 mutation increases Sox10-dependent hypopigmentation and does not result in dark skin. (A, B) Rps7Zma/+; Sox10LacZ/+ double mutants that survive postnatally exhibit more extensive hypopigmentation than Rps7Zma/+or Sox10LacZ/+ mutants. A representative Rps7Zma/+; Sox10LacZ/+ mouse is shown (A) along with quantitative scoring (0 = no spotting; 4 = the largest ventral spots that extend to the dorsal surface) comparing the white spotting in Rps7+/+; Sox10LacZ/+ (blue, N = 27), Rps7Zma/+; Sox10+/+ (red, N = 16), and Rps7Zma/+; Sox10LacZ/+ (yellow, N = 8) (B). (C, D) Adult Rps7 mutants do not have dark skin in foot pads and tails. (E,F) H&E stained sections through the tail skin of Rps7Zma/+ mice reveal epidermal (*) and dermal (arrowhead) pigmentation similar to that of Rps7+/+ mice, confirming the presence of melanocytes in the tail. (TIF) [file pgen.1003094.s007.tif]

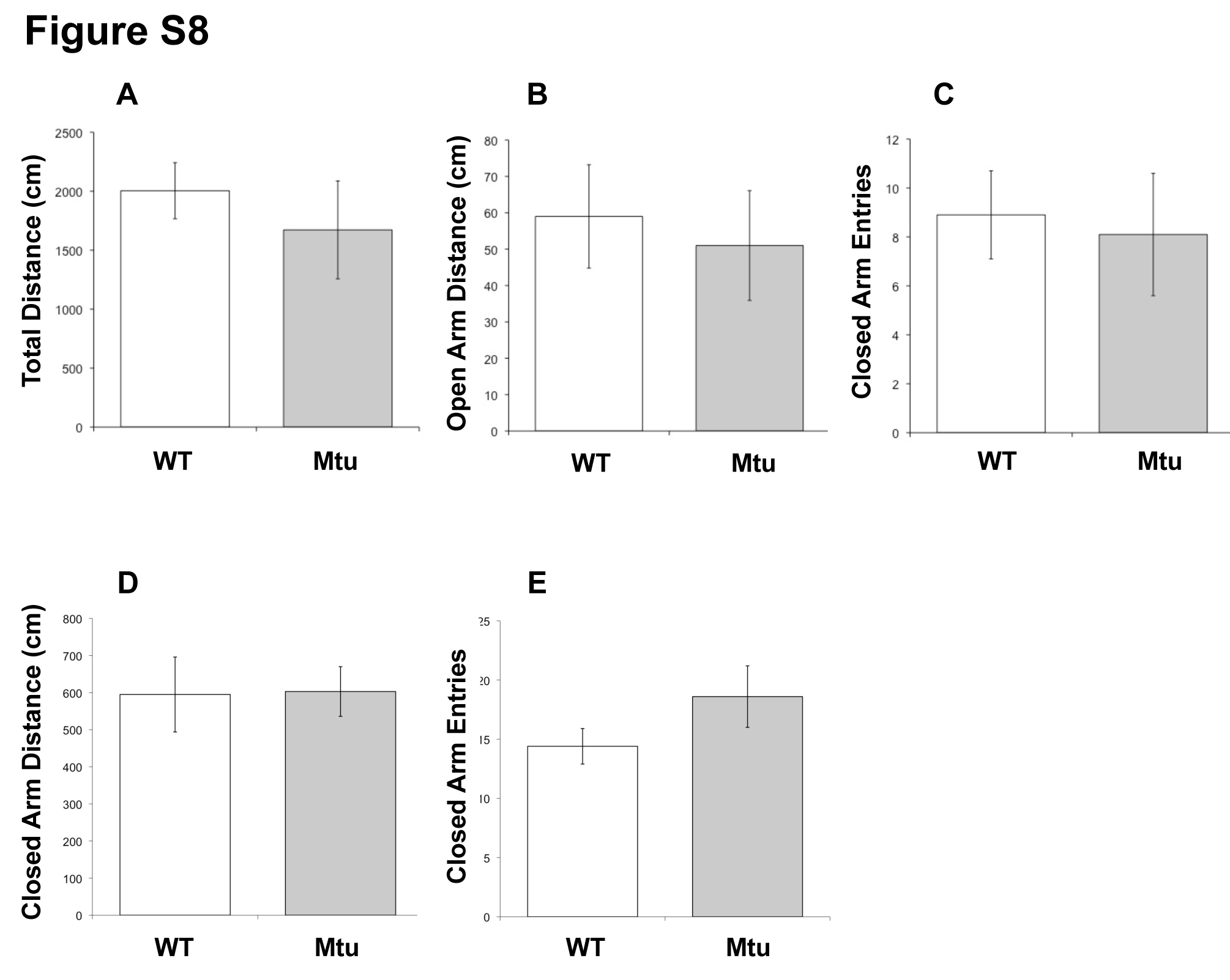

Supplement: Figure S8 — Behavioral assessment of Rps7Mtu/+ mutants. (A) Total distance traveled in the open-field test by Rps7+/+ controls (WT) and Rps7Mtu/+ (Mtu) (N = 11) was similar (p = 0.5). (B–E) Likewise there were no significant differences between Rps7+/+ and Rps7Mtu/+ on the elevated plus maze. Open arm distance (B, P>0.5), open arm entries (C, P>0.5), closed arm distance (D, P>0.5), and closed arm entries (E, P>0.1) were all comparable (N = 11). (TIF) [file pgen.1003094.s008.tif]

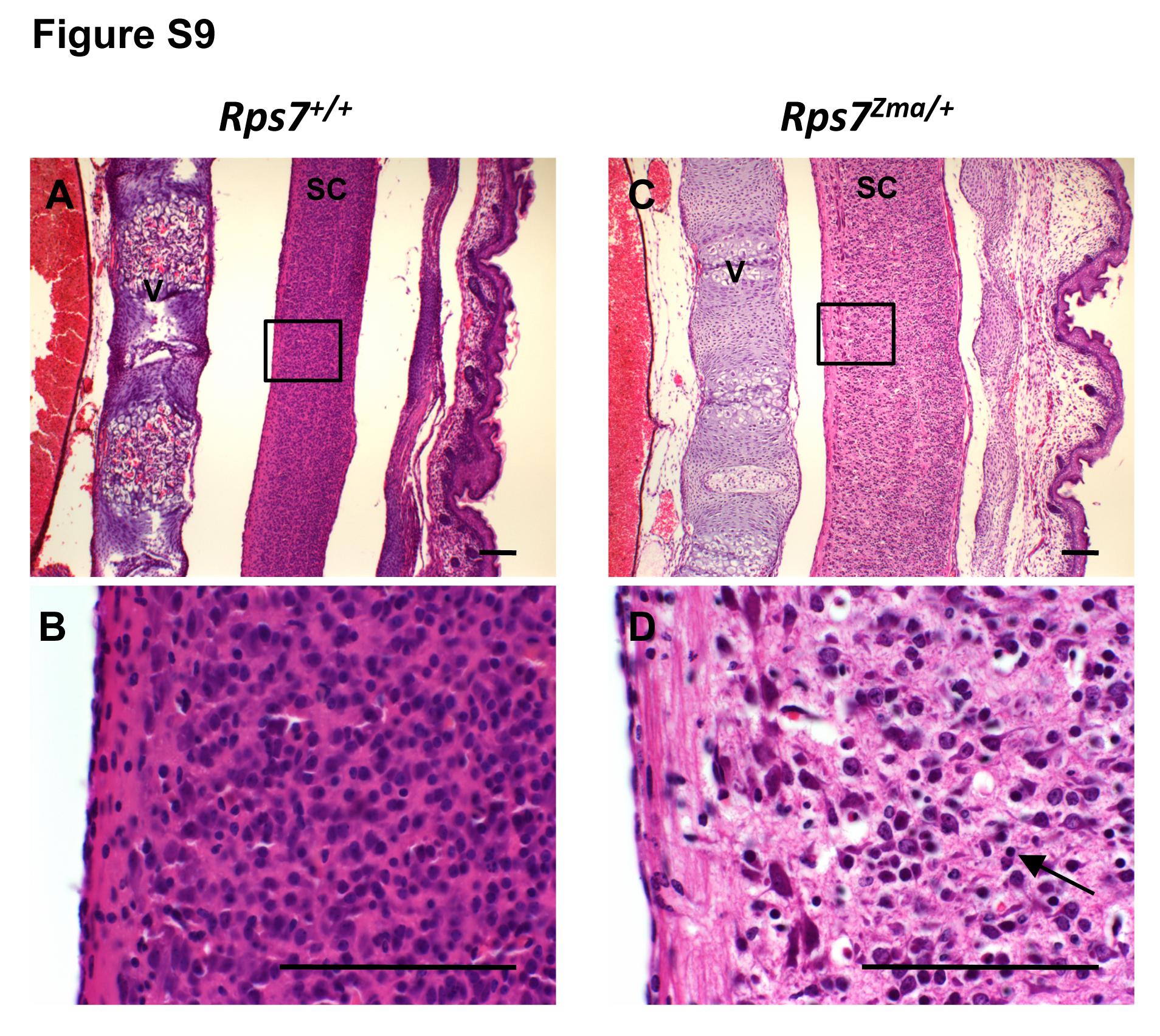

Supplement: Figure S9 — Rps7 mutants have pyknotic nuclei in the spinal cord. H&E stained sagittal sections through the spinal cord of E18.5 Rps7+/+ (A, B) and Rps7Zma/+ (C, D) embryos. Arrow in D indicates an example of a darkly stained, condensed nucleus characteristic of pyknotic nuclei observed throughout the Rps7Zma/+ spinal cord. Anterior is oriented towards the top and posterior to the bottom of all images. Boxed regions in A, C define the respective areas shown at higher magnification in B, D. Abbreviations: vertebral column (V), spinal cord (SC). Scale bars = 100 µm. (TIF) [file pgen.1003094.s009.tif]

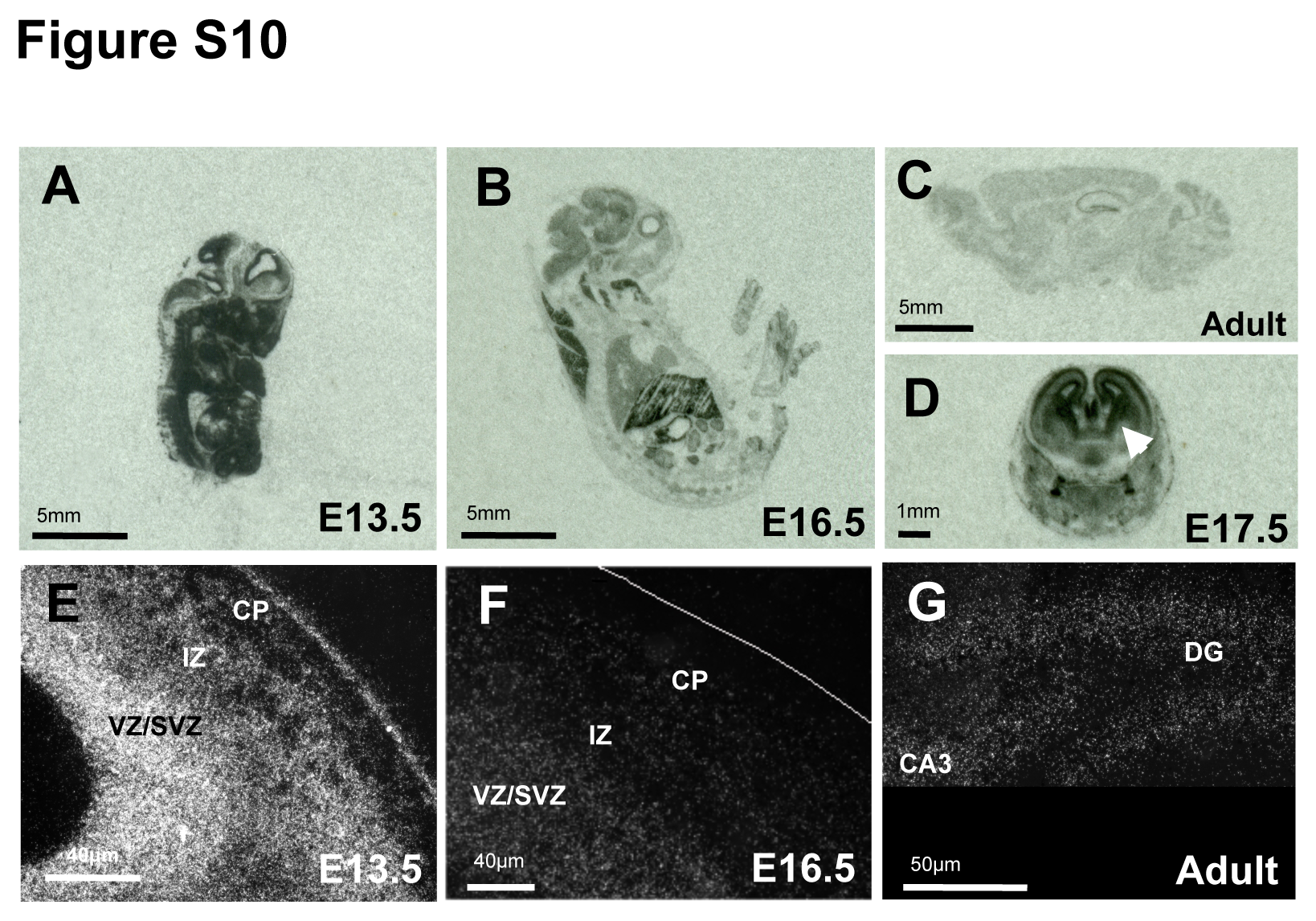

Supplement: Figure S10 — Rps7 mRNA expression pattern. Mouse Rps7 transcript was detected by in situ hybridization at E13.5 (A, E), E16.5 (B, F), E17.5 (D), and adulthood (C, G). Broad expression was detected in the developing embryo, including a high level of expression in the ganglionic eminences (arrowhead in D), the proliferative ventricular zone (E), as well as in the dentate gyrus on the hippocampus in adult animals (G). Abbreviations: ventricular zone/subventricular zone (VZ/SVZ), intermediate zone (IZ), cortical plate (CP), CA3 region of hippocampus (CA3), dentate gyrus (DG). (TIF) [file pgen.1003094.s010.tif]

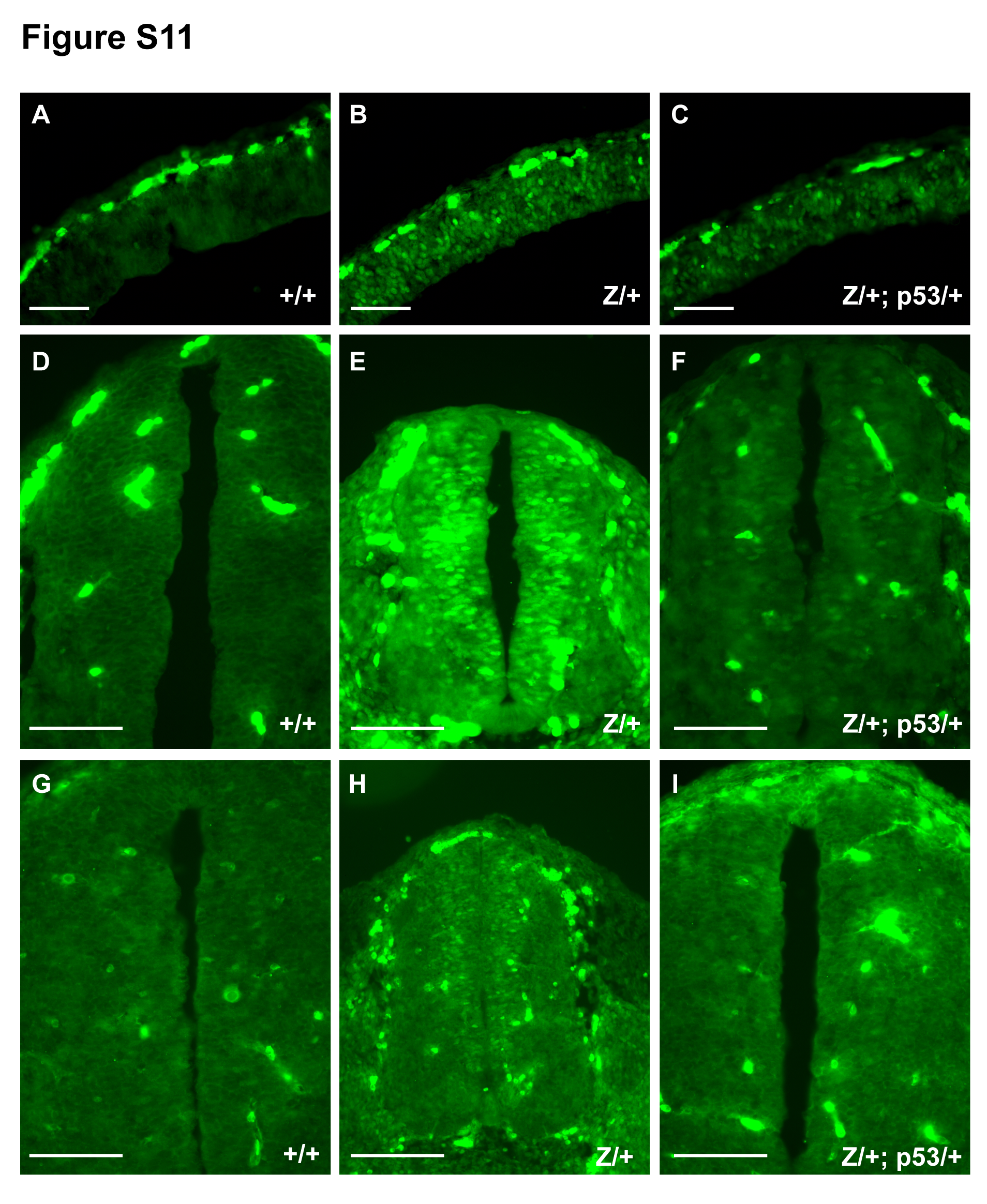

Supplement: Figure S11 — Rps7 mutants have increased TRP53 expression. (A–C) Increased TRP53 expression was detected in E11.5 Rps7Zma/+ (Z/+) coronal sections through the neocortex compared to Rps7+/+ (+/+) and Rps7Zma/+; Trp53KO/+ (Z/+;p53/+). (D–I) This increase was also detected in the neural tube of Rps7Zma/+ embryos at E11.5 (D–F) and E12.5 (G–I). Scale bars = 100 µm. (TIF) [file pgen.1003094.s011.tif]
