# Supplementary material for: Mutation of the Diamond-Blackfan Anemia Gene Rps7 in Mouse Results in Morphological and Neuroanatomical Phenotypes
Source: PLoS Genet. 2013 Jan 31;9(1):e1003094. doi: 10.1371/journal.pgen.1003094 (PMC3561062; doi:10.1371/journal.pgen.1003094)
Supplement: Table S1 — Circulating blood counts from+/+and Rps7Mtu/+ adult male mice. (PDF) [file pgen.1003094.s012.pdf]

Table S1. Circulating blood counts from +/+ and *Rps7*^{Mtu}/+ adult male mice (N = 5).

		Wild-type (*Rps7*+/+)		Heterozygote (*Rps7*^{Mtu}/+)	
**Parameter**	**Unit**	**Average**	**StDev**	**Average**	**StDev**
**WBCP**	x10E03 cells/μL	5.2	1.1	4.5	1.2
**WBCB**	x10E03 cells/μL	5.1	1.0	4.5	1.2
**RBC**	x10E06 cells/μL	9.5	0.6	9.0	0.7
**measHGB**	g/dL	14.7	0.9	14.4	0.8
**HCT**	L/L	0.5	0.0	0.4	0.0
**MCV**	fL	49.2	0.6	50.5	0.3
**MCH**	pg	15.4	0.3	15.8	0.6
**MCHC**	g/dL	31.3	0.8	31.2	1.1
**CHCM**	g/dL	30.8	1.2	30.4	0.7
**RDW**	%	12.7	1.1	12.8	0.6
**HDW**	g/dL	2.2	0.3	2.0	0.2
**PLT**	x10E03 cells/μL	1183.6	108.3	1210.2	122.4
**MPV**	fL	7.2	0.8	7.1	1.1
**PDW**	%	60.3	4.7	55.8	5.0
**PCT**	%	0.8	0.1	0.8	0.2
**NEUT**	%	22.3	8.9	21.2	8.7
**LYM**	%	71.2	8.4	72.2	9.6
**MONO**	%	3.2	1.2	4.1	2.2
**EOS**	%	2.6	1.3	2.2	1.0
**LUC**	%	0.6	0.4	0.3	0.2
**BASO**	%	0.1	0.2	0.1	0.1
**ABS Neutros**	x10E03 cells/μL	1.1	0.6	1.1	0.7
**ABS Lymphs**	x10E03 cells/μL	3.6	0.8	3.1	0.9
**ABS Monos**	x10E03 cells/μL	0.2	0.1	0.2	0.0
**ABS Eos**	x10E03 cells/μL	0.1	0.1	0.1	0.1
**ABS Lucs**	x10E03 cells/μL	0.0	0.0	0.0	0.0
**ABS Basos**	x10E03 cells/μL	0.0	0.0	0.0	0.0
**Retic**	x10E9 cells/L	285.6	32.6	289.9	30.1
**Retic**	%	2.2	1.5	2.3	1.6
**PLT**	x10E03 cells/μL	1183.6	108.3	1210.2	122.4
**Large PLT**	x10E03 cells/μL	15.3	7.6	14.0	12.5
