# Supplementary material for: Mutation of the Diamond-Blackfan Anemia Gene Rps7 in Mouse Results in Morphological and Neuroanatomical Phenotypes
Source: PLoS Genet. 2013 Jan 31;9(1):e1003094. doi: 10.1371/journal.pgen.1003094 (PMC3561062; doi:10.1371/journal.pgen.1003094)
Supplement: Table S2 — Biochemical panel from+/+and Rps7Mtu/+ adult male mice. (PDF) [file pgen.1003094.s013.pdf]

Table S2. Biochemical panel from +/+ and *Rps7*^{Mtu}/+ adult male mice (N = 9).

	Wild-type *Rps7*+/+		Heterozygote *Rps7*^{Mtu}/+	
**Parameter**	**Average**	**StDev**	**Average**	**StDev**
**Sodium** (mmol/l)	150.7	1.6	151.0	0.9
**Potassium** (mmol/l)	5.1	1.1	5.7	0.9
**Chloride** (mmol/l)	106.8	3.1	108.3	2.0
**Urea** (mmol/l)	9.3	1.8	10.1	1.5
**Creatinine (Enzymatic)** (µmol/l)	11.7	3.3	12.8	1.4
**Calcium** (mmol/l)	2.5	0.1	2.5	0.1
**Phosphorus** (mmol/l)	2.1	0.6	2.5	0.3
**ALP** (U/l)	102.0	23.3	111.8	12.9
**ALT** (U/l)	42.0	17.2	53.1	31.1
**AST** (U/l)	60.1	11.5	62.6	24.5
**Tot. Protein** (g/l)	52.8	3.2	52.0	4.1
**Albumin** (g/l)	26.4	1.5	25.7	2.5
**Tot. Cholesterol** (mmol/l)	3.8	0.7	3.1	1.1
**Glucose** (mmol/l)	12.4	3.9	11.9	2.4
**Triglycerides** (mmol/l)	3.8	1.8	3.5	1.8
**Glycerol** (µmol/l)	396.2	124.7	429.1	210.3
**FFA** (mmol/l)	1.0	0.3	1.1	0.6
**LDH** (U/l)	539.7	217.9	798.2	419.8
**Iron** (µmol/l)	38.5	4.4	42.6	3.5
**Amylase** (U/l)	679.5	87.3	610.3	36.0
