# Supplementary material for: Mutation of the Diamond-Blackfan Anemia Gene Rps7 in Mouse Results in Morphological and Neuroanatomical Phenotypes
Source: PLoS Genet. 2013 Jan 31;9(1):e1003094. doi: 10.1371/journal.pgen.1003094 (PMC3561062; doi:10.1371/journal.pgen.1003094)
Supplement: Table S3 — Rps7Zma/+ erythrocytes are able to terminally differentiate, but they display modest developmental delay that is suppressed by Trp53 haploinsufficiency. (PDF) [file pgen.1003094.s014.pdf]

Table S3. *Rps7*^{Zma}/+ erythrocytes are able to terminally differentiate, but they display modest developmental delay that is suppressed by *Trp53* haploinsufficiency.

	Rps7+/+		***Rps7***^{Zma}***/+***		***Rps7***^{Zma}***/+; Trp53 +/-***		***Trp53+/-***	
	**E13.5 (N=6)**	**E14.5 (N=2)**	**E13.5 (N=6)**	**E14.5 (N=6)**	**E13.5 (N=2)**	**E14.5 (N=6)**	**E13.5 (N=5)**	**E14.5 N=8)**
**R1**	4.8±1.60*	5.3±1.47	3.44±1.64	12.7±4.14[2]	4.0±1.48	6.9±3.54	5.7±0.79	3.7±1.47
**R2**	16.4±4.00	2.8±0.31	15.62±3.96	5.2±0.51	12.6±9.60	4.5±2.53	15.6±4.53	4.6±1.47
**R3**	69.8±2.04	66.5±0.57	74.83±1.28[1]	59.8±5.40	72.3±0.99	56.5±4.98	68.9±2.08	58.6±4.79
**R4**	6.2±3.05	2.5±0.15	3.83±2.66	2.9±0.42	7.5±5.42	2.4±0.62	6.7±3.49	2.8±1.62
**R5**	0.2±0.15	1.5±0.05	0.09±0.11	1.5±0.28	0.8±1.07	1.6±0.12	0.2±0.11	1.8±0.29

*Values are presented as % of cells in each population
Gray = populations where *Rps7*^{Zma}/+sample varied significantly from age-matched *Rps7+/+* or *Rps7*^{Zma}*/+; Trp53 +/-* by ANOVA with post-hoc analysis and Bonferroni p-value correction.

[1] p<0.001 *Rps7+/+* vs *Rps7*^{Zma}/+at E13.5

[2] p<0.05 for *Rps7+/+* vs *Rps7*^{Zma}/+ and for *Rps7*^{Zma}/+ vs *Rps7*^{Zma}*/+; Trp53+/-* at E14.5
