# Supplementary material for: Mutation of the Diamond-Blackfan Anemia Gene Rps7 in Mouse Results in Morphological and Neuroanatomical Phenotypes
Source: PLoS Genet. 2013 Jan 31;9(1):e1003094. doi: 10.1371/journal.pgen.1003094 (PMC3561062; doi:10.1371/journal.pgen.1003094)
Supplement: Table S4 — Surviving numbers of postnatal offspring from an Rps7Zma/+; Trp53+/−×C57BL/6J cross demonstrate that Trp53 haploinsufficiency restores Rps7 viability. (PDF) [file pgen.1003094.s015.pdf]

Table S4. Surviving numbers of postnatal offspring from an *Rps7*$^{Zma/+}$; *Trp53*$^{+/-}$ x C57BL/6J cross demonstrate that *Trp53* haploinsufficiency restores *Rps7* viability.

	Rps7$^{+/+}$		***Rps7***$^{Zma/+}$	
	Trp53$^{+/+}$	***Trp53***$^{+/-}$	***Trp53***$^{+/+}$	***Trp53***$^{+/-}$
E11.5	18 (31%)	16 (28%)	13 (22%)	11 (19%)
E12.5	16 (20%)	22 (27%)	20 (25%)	23 (28%)
E13.5	7 (32%)	7 (32%)	6 (27%)	2 (9%)
E14.5	11 (17%)	20 (31%)	11 (17%)	23 (35%)
E18.5	23 (24%)	29 (31%)	25 (27%)	17 (18%)
P0	4 (50%)	1 (13%)	0 (0%)	3 (38%)
P2	8 (44%)	4 (22%)	0 (0%)	6 (33%)
P5	1 (20%)	1 (20%)	0 (0%)	3 (60%)
Weaning	17 (33%)	12 (23%)	0 (0%)	22 (43%)
Expected Frequency	25%	25%	25%	25%
